# Four new species of *Pristimantis myersi* group (Anura: Craugastoridae) from Carchi, Ecuador: cryptic diversity in unique and threatened high-Andean ecosystems

**DOI:** 10.7717/peerj.21618

**Published:** 2026-08-26

**Authors:** Daniela Franco-Mena, Mateo A. Vega-Yánez, Malki Bustos, Juan M. Guayasamin, Diego Batallas

**Affiliations:** 1 Laboratorio de Biología Evolutiva, Global Research & Solutions Center, Colegio de Ciencias Biológicas y Ambientales, Universidad San Francisco de Quito USFQ, Quito, Campus Cumbayá, Pichincha, Ecuador; 2 Tandayapa Cloud Forest Station, Colegio de Ciencias Biológicas y Ambientales, Universidad San Francisco de Quito USFQ, Quito, Ecuador; 3 Instituto Nacional de Biodiversidad, Quito, Ecuador; 4 Departamento de Biodiversidad, Ecología y Evolución de la Facultad de Ciencias Biológicas, Universidad Complutense de Madrid, Madrid, Spain

**Keywords:** Conservation, Endemism, New species, *Pristimantis*, Rain frogs, Tropical Andes

## Abstract

The high-Andean ecosystems of the northern Andes rank among the most diverse and endemic regions on the planet. This study describes four new species of frogs in the genus *Pristimantis*, belonging to the *myersi* group. The four new species were discovered through 6 years of fieldwork (2016–2021) as part of doctoral research integrating morphological, bioacoustic, ecological, and molecular data. Phylogenetic analyses based on mitochondrial DNA (mtDNA) support the recognition of each taxon as a distinct species of *Pristimantis*, known for its cryptic diversity and restricted distribution. Additionally, we document changes in vegetation cover across the habitats where these species occur, underscoring the fragility of their ecosystems. This study represents the first attempt to link the distribution of these species with the spatiotemporal dynamics of vegetation cover in Carchi, showing a net loss of more than 9,000 hectares between 1985 and 2022, particularly in the western Andean foothills. Finally, our findings uncover the previously undocumented biological richness of Carchi Province and highlight the urgent need to conserve its unique and threatened high-Andean ecosystems.

## Introduction

The province of Carchi, located in the northern Ecuadorian Andes, occupies a unique biogeographic position between the eastern and western branches of the Andean Mountain range. This unique environment has led to a remarkable level of biological diversity, marked by a high degree of endemism (Yánez-Muñoz et al., 2020). Among its ecosystems, high Andean habitats are the most representative, encompassing montane forests and extensive expanses of páramo (Batallas, Márquez & Guayasamin, 2025). These environments support frog assemblages predominantly composed of species from the genus *Pristimantis* Jiménez de la Espada, 1870. This genus of terrestrial frogs with presumed direct development currently comprises 637 species (Frost, 2026), making it by far the most species-rich amphibian genus worldwide (Heinicke, Duellman & Hedges, 2007; Hedges, Duellman & Heinicke, 2008; Padial, Grant & Frost, 2014; Waddell et al., 2018; Acevedo-Rincón, Palma & Olalla-Tárraga, 2022).

Given the significant diversity of species in the genus *Pristimantis*, numerous phenetic groups have been defined with the aim of facilitating their taxonomic management (Lynch, 1976a; Lynch & Duellman, 1997; Hedges, Duellman & Heinicke, 2008; Pinto-Sánchez et al., 2012; Ortega, Brito & Ron, 2022). One such group is the *Pristimantis myersi* species group (Hedges, Duellman & Heinicke, 2008; Taboada et al., 2013), characterized by small sized individuals (SVL < 34.6 mm), robust bodies, short snouts, limbs short to moderate long, finger I shorter than finger II, toe V slightly longer than toe III and not extending to the proximal edge of the distal subarticular tubercle of the toe IV, and digital discs narrow (expanded in *P. floridus*) and rounded (lanceolate in *P. hectus*) (Lynch & Duellman, 1997; Hedges, Duellman & Heinicke, 2008; Franco-Mena et al., 2023; Yánez-Muñoz et al., 2025).

The *Pristimantis myersi* species group is distributed throughout the Andean regions of Ecuador and southern Colombia. According to the most recent taxonomic and phylogenetic studies, 28 species are currently recognized within the group (Franco-Mena et al., 2023; Reyes-Puig et al., 2023; Yánez-Muñoz et al., 2025). However, this number should be considered provisional, as the group exhibits considerable cryptic and polymorphic diversity, with several candidate species collected during various studies that have yet to be formally described (Franco-Mena et al., 2023; Yánez-Muñoz et al., 2025).

In this context, the province of Carchi, particularly its high Andean ecosystems, represents a region with a significant gap in knowledge regarding its herpetological diversity, underscoring the need for integrative approaches to accurately delimit species (Batallas, Márquez & Guayasamin, 2025). As part of doctoral research (Diego Batallas), which involved numerous expeditions aimed at documenting amphibian diversity. Herein we describe four of the new candidate species of the *Pristimantis myersi* group, using an integrative approach that combines morphological, acoustic and molecular evidence, these species were discovered in the high Andean ecosystems of Carchi Province, Ecuador (Franco-Mena et al., 2023; Batallas, Márquez & Guayasamin, 2025).

## Materials and Methods

**Figure 1:**
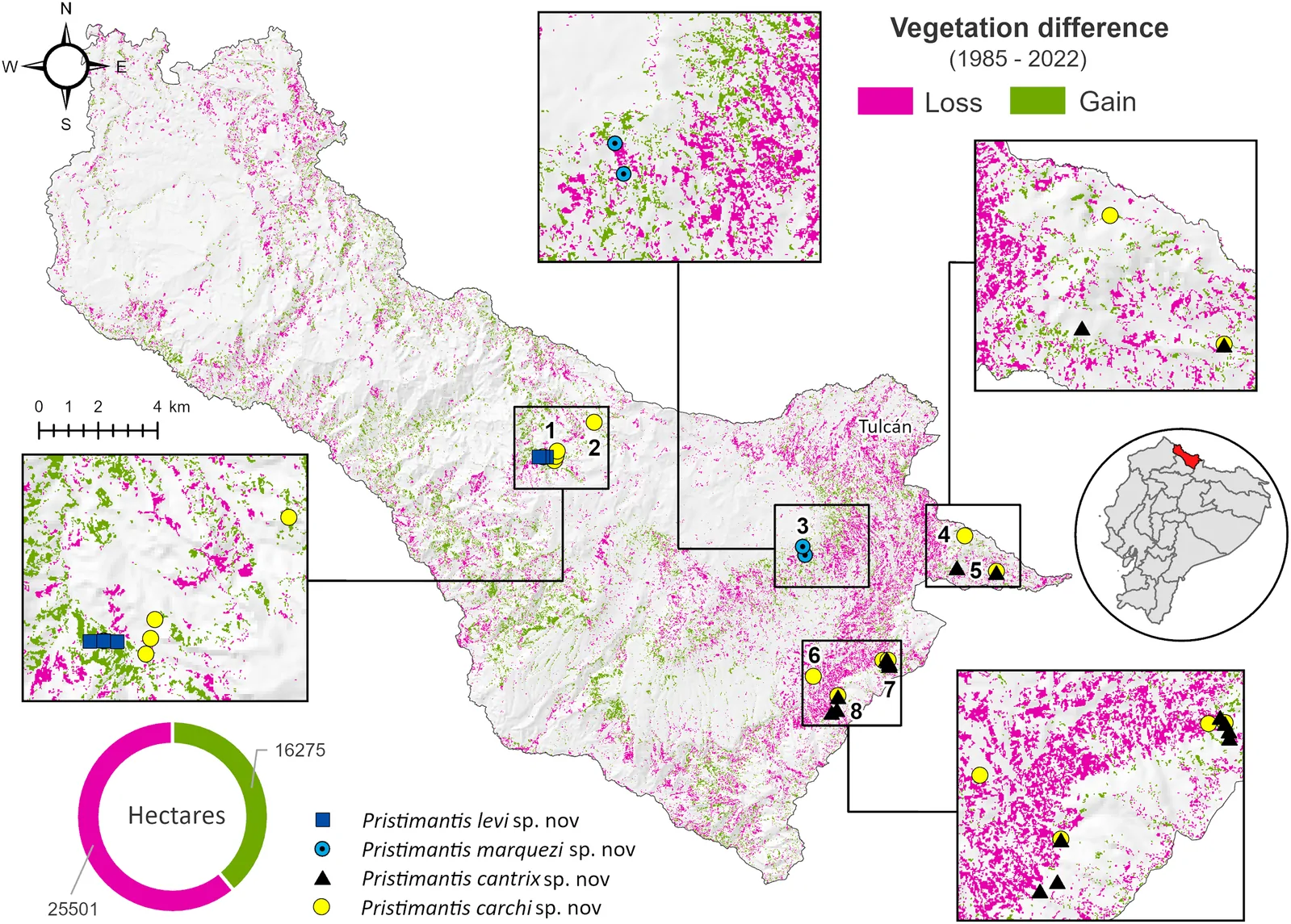
Distribution of new species and spatial-temporal dynamics of vegetation in the province of Carchi. The numbers represent the localities: (1) Morán, (2) Camino Tufiño-Maldonado, (3) San Francisco de Pioter, (4) Cascadas de Cartagena, (5) Virgen Negra, (6) Bosque de los Arrayanes, (7) Cerro La Bretaña, and (8) Loma La Esperanza. Prepared by Mateo A. Vega-Yánez.

**Study area.** Fieldwork comprised multiple expeditions conducted within the high Andean ecosystems of Carchi Province, northern Ecuador. These expeditions were part of the field component of a doctoral research project (2016–2021) that focused on the diversity and acoustic ecology of amphibians inhabiting these environments. The four new species were collected from 13 localities distributed along both the western and eastern slopes of the Andean Mountain range, at elevations ranging from 2,694 to 3,848 m (Fig. 1).

**Ethics statement.** Specimens were euthanized following the ethical guidelines of Chen & Combs (1999) and preserved according to the protocols outlined by Simmons (2015). Liver tissues were extracted for genetic analyses and stored in 99% ethanol. All specimen handling and collection were conducted under permits issued by the Ministerio del Ambiente y Energía (MAE-DNB-CM-2016-0045 and MAE-DNB-CM-2018-0105). Voucher specimens were deposited at the Museo de Zoología of Universidad San Francisco de Quito (ZSFQ), and tissue samples for molecular analyses are housed at the Laboratorio de Biología Evolutiva (LBE) of the same university.

**Morphological data and nomenclature.** The species description adheres to the standards set by Lynch & Duellman (1997), with diagnostic characteristics defined by Duellman & Lehr (2009) and Ospina-Sarria & Duellman (2019). For the systematic classification of the family, we adopted the framework proposed by Heinicke et al. (2017). Additionally, we followed the species groups suggested by Hedges, Duellman & Heinicke (2008) and Padial, Grant & Frost (2014), Franco-Mena et al. (2023) and Yánez-Muñoz et al. (2025). The morphometric measures were taken with an electronic caliper Mitutoyo (precision ± 0.01 mm, rounded to 0.1 mm) following the comments of Lynch & Duellman (1997) and Duellman & Lehr (2009): snout-vent length (SVL), head length (HL), head width (HW), interorbital distance (IOD), upper eyelid width (UEW), eye length (EL), eye-nostril distance (END), tympanic annulus (TY), internarinal distance (IND), upper arm length (UAL), forearm length (FLL), hand length (HAL), Finger I; Finger II; Finger III disk width (Finger III DW); thigh length (THL); tibia length (TL); foot length (FL); and Toe IV disk width (Toe IV DW).

The electronic version of this article in Portable Document Format (PDF) will represent a published work according to the International Commission on Zoological Nomenclature (ICZN), and hence the new names contained in the electronic version are effectively published under that Code from the electronic edition alone. This published work and the nomenclatural acts it contains have been registered in ZooBank, the online registration system for the ICZN. The ZooBank LSIDs (Life Science Identifiers) can be resolved and the associated information viewed through any standard web browser by appending the LSID to the prefix http://zoobank.org/. The LSID for this publication are: [urn:lsid:zoobank.org:act:20F3BD35-F442-459A-BBAE-D8C4352B6EE9, urn:lsid:zoobank.org:act:3148008B-EFE2-4146-B5A6-F1702444135C, urn:lsid:zoobank.org:act:19332353-1131-421C-86B8-AD0E79E9986A, and urn:lsid:zoobank.org:act:4472300F-D4EA-4A64-8571-249E3C3C1077]. The online version of this work is archived and available from the following digital repositories: PeerJ, PubMed Central SCIE and CLOCKSS.

### Analysis of data

**Morphometry and bioacoustics.** To evidence the morphological differences between the new species based on the nineteen body measurements, and to reduce the effects of allometry in the analyses (Klingenberg, 2016), we performed a linear regression between the SVL (Snout-Vent Length) and each body measurement, using the RRPP package version 1.4.0. in R (Collyer & Adams, 2024). With the residuals obtained we performed a principal component analysis (PCA) and a discriminant analysis of principal components (DAPC), with the adegenet package version 2.1.10 in R (Jombart, 2008). These analyses enabled the clustering and differentiation of species, and to increase the evidence for differences among the new species, we analyzed thirty acoustic parameters. First, the data were normalized: they were centered by subtracting the mean and scaled by dividing by the standard deviation. From these normalized data, PCA and DAPC were generated in R software (R Core Team, 2024). For bioacoustics, we based on results in Batallas, Márquez & Guayasamin (2025).

**Phylogenetic relationships.** Recent sampling efforts and the mitochondrial phylogeny by Franco-Mena et al. (2023) have provided an initial insight into the biogeography and speciation patterns of the *Pristimantis myersi* group, including the new species described in this work. We inferred the evolutionary relationships and genetic distances with the same data matrix and criteria described by Franco-Mena et al. (2023).

**Vegetation analysis.** To estimate the amount of vegetation lost in the province of Carchi between 1985–2022, we performed a spatio-temporal analysis of vegetation cover. We downloaded land cover and land use data from the MapBiomas platform (available at http://ecuador.mapbiomas.org). These data provide information with a spatial resolution of 30 m and were generated by supervised classification of satellite images. Based on this geospatial information, first, using the sf package in R (Pebesma, 2018), the coverages were converted to the spatial reference system WGS 1984 UTM Zone 17S to ensure a correct superposition and comparison of the geographic information. Subsequently, using the st_difference function, the values of vegetation loss/gain in the province were calculated. Also, a limitation is that some greenery may not be actual forest, but just green cover of agriculture or similar land uses which may not be suitable for species to occur.

## Results

**Figure 2:**
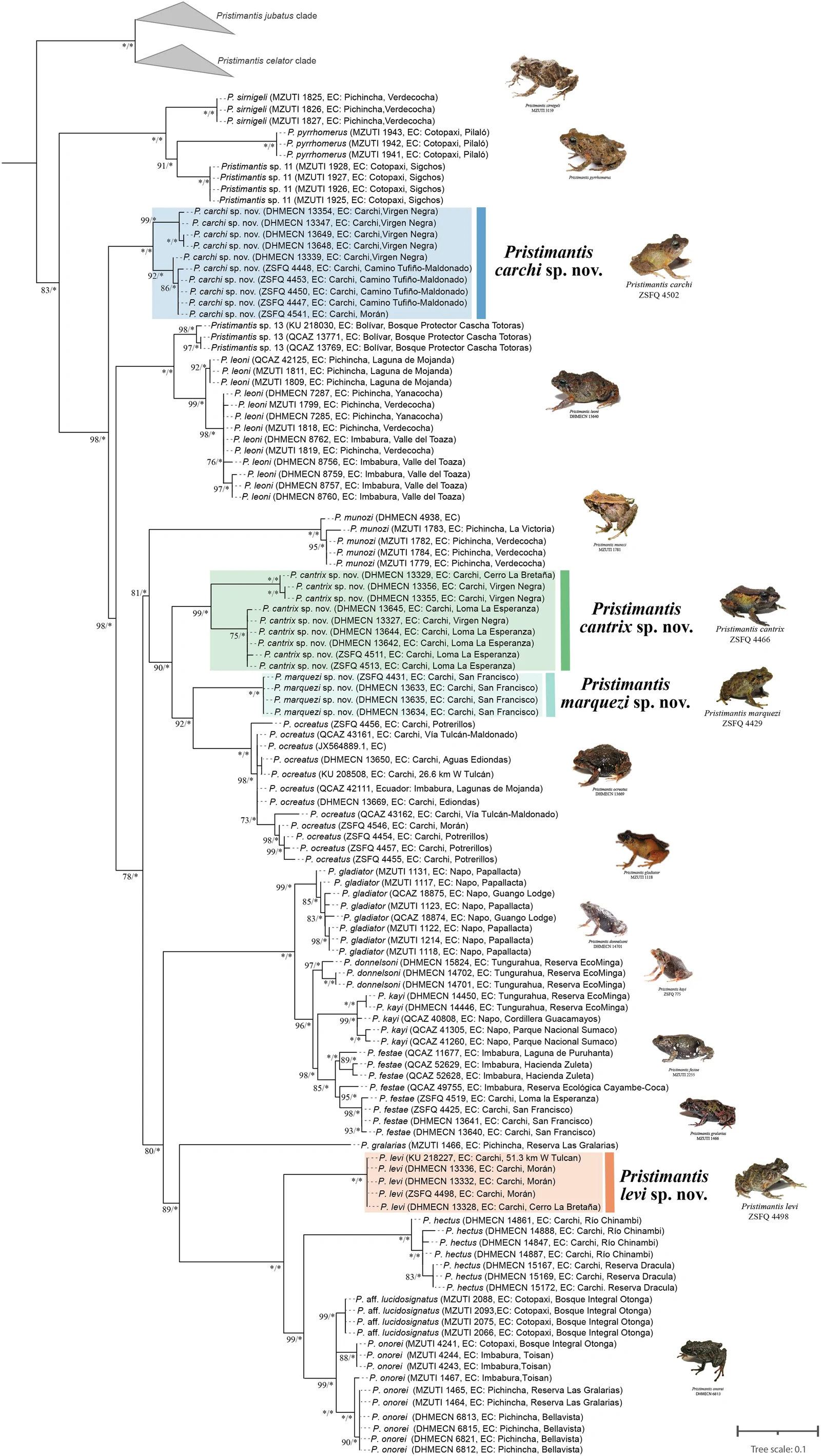
Evolutionary relationships amongst species in the *Pristimantis myersi* group. Under the Maximum Likelihood inference criterion modified from Franco-Mena et al. (2023). Prepared by Daniela Franco-Mena.

**Generic placement.** We assign the four new species in *Pristimantis* based on morphology, acoustic and molecular data (Hedges, Duellman & Heinicke, 2008; Taboada et al., 2013; Franco-Mena et al., 2023; Batallas, Márquez & Guayasamin, 2025). The inferred evolutionary relationships shown in Fig. 2 are the same as those published by Franco-Mena et al. (2023), the new species described below represent Candidate Species 12, 14, 15, and 18 reported by Franco-Mena et al. (2023).

### New species

***Pristimantis cantrix sp. nov***

*Pristimantis* sp. 14, Franco-Mena et al., 2023

*Pristimantis* sp. 3 Batallas, Márquez & Guayasamin, 2025

**LSID.** urn:lsid:zoobank.org:act:20F3BD35-F442-459A-BBAE-D8C4352B6EE9

**Proposed standard English name:** Cantor Rain Frog

**Proposed standard Spanish name:** Cutín cantor de Carchi

**Figure 3:**
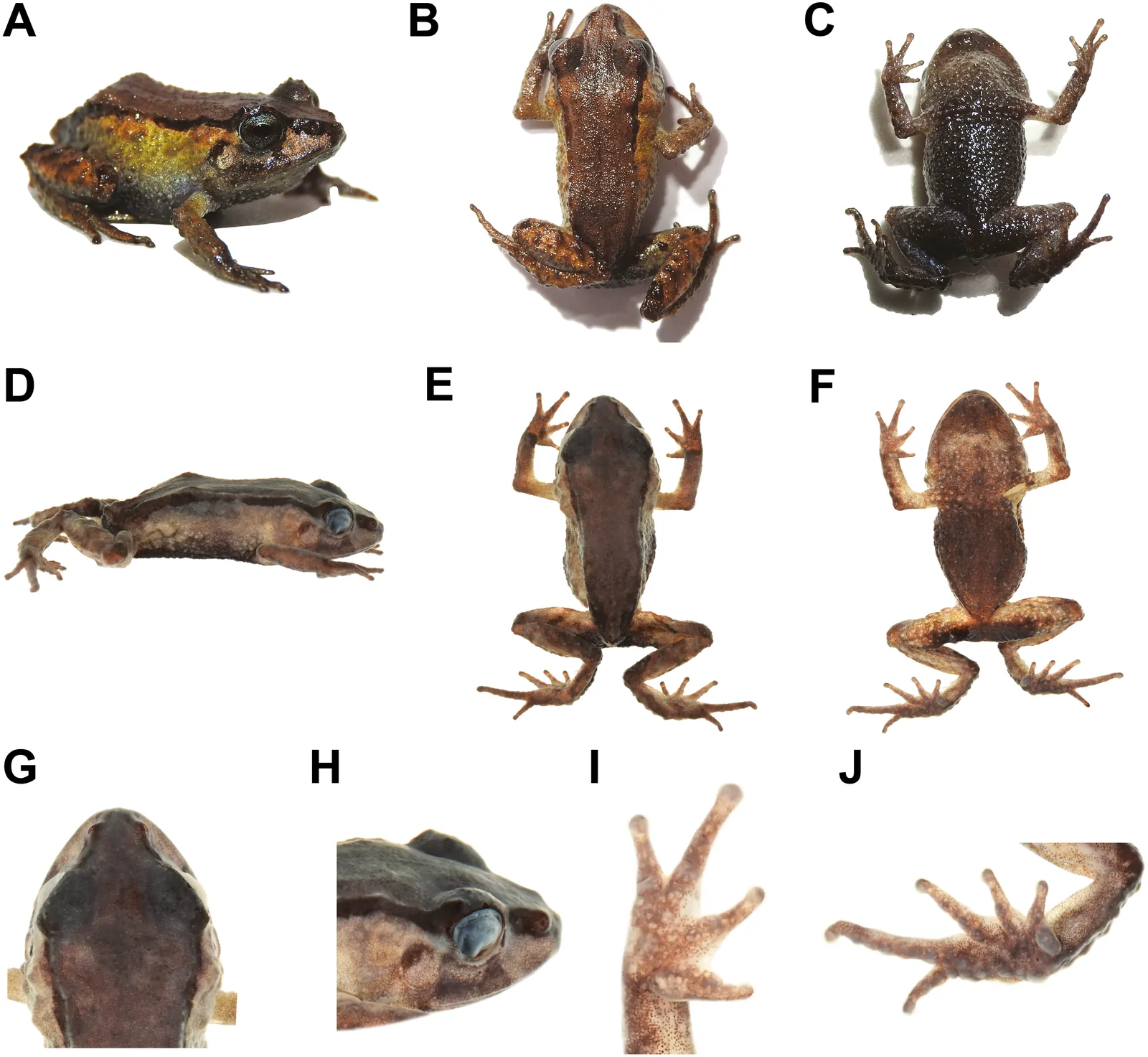
Holotype of *Pristimantis cantrix* sp. nov. (ZSFQ 4466), adult male, SVL = 14.4 mm. (A–C) Live and (D–F) preserved photographs in lateral, dorsal and ventral view; (G) head in dorsal and (H) lateral view, (I) palmar surface, and (J) plantar surface. Photographs by Diego Batallas, Malki Bustos, and Daniela Franco-Mena.

**Holotype (Fig. 3).** ZSFQ 4466 (adult male), collected on November 10, 2020, by Diego Batallas, and Marcelo Oliva, at Virgen Negra (0.659583 N, 77.645 W; 3,612 m), Carmelo, Tulcán, Carchi province, Ecuador.

**Paratypes.** A total of 37 specimens, including adult males (32) and females (5), were collected between February 2020 and January 2021 across three localities in Carchi province, Ecuador. ZSFQ 4467 (male), with same data as the holotype. ZSFQ 4469 (male), ZSFQ 4471 (male), with same data as the holotype, collected on 12 November 2020. ZSFQ 4472 (female), ZSFQ 4473 (male), with same data as the holotype, collected on 13 November 2020. ZSFQ 4475 (male), with same data as the holotype, collected on 14 November 2020. ZSFQ 4451 (female), ZSFQ 4552, 4553 (males), collected on 21 February 2020. ZSFQ 4554 (male), ZSFQ 4555 (female), collected on 22 February 2020. ZSFQ 4556, 4557 (males), collected on 23 February 2020 by Diego Batallas, Gabriela Olmedo and Jaime Rosero at Cerro La Bretaña (0.5625 N, 77.711917 W; 3,447 m), Fernández Salvador, Montúfar, Carchi province, Ecuador. ZSFQ 4558, 4559 (males), ZSFQ 4560 (female), collected on 24 February 2020. ZSFQ 4562, 4563 (males), collected on 25 February 2020 by Diego Batallas, Gabriela Olmedo and Jaime Rosero at Cerro La Bretaña (0.564694 N, 77.712056 W; 3,372 m), Fernández Salvador, Montúfar, Carchi province, Ecuador. ZSFQ 4564, 4566, 4567, 4568, 4569 (males), collected on 27 February 2020. ZSFQ 4575 (male), collected on 29 February 2020 by Diego Batallas, Gabriela Olmedo and Jaime Rosero at Cerro La Bretaña (0.567 N, 77.71325 W; 3,255 m), Fernández Salvador, Montúfar, Carchi province, Ecuador. ZSFQ 4508, 4509 (males), collected on 12 January 2021. ZSFQ 4510 (male), ZSFQ 4511 (female), collected on 14 January 2021 by Diego Batallas, Roque Cerón and Luis Cuasquer at Loma La Esperanza (0.518806 N, 77.764028 W; 3,019 m), San José-Chamizo, Montúfar, Carchi province, Ecuador. ZSFQ 4512, 4513, 4514 (males), collected on 15 January 2021. ZSFQ 4517, 4518 (males), collected on 16 January 2021. ZSFQ 4520, 4521, 4522, 4523 (males), collected on 17 January 2021 by Diego Batallas, Roque Cerón and Luis Cuasquer at Loma La Esperanza (0.515999 N, 77.769306 W; 3,118 m), San José-Chamizo, Montúfar, Carchi province, Ecuador.

**Diagnosis.** *Pristimantis cantrix* sp. nov. can be distinguished from other *Pristimantis* by the following character combination: (1) skin on dorsum finely tuberculate, with scattered larger tubercles; low sinusoidal, W, or )( shaped scapular ridges, aligned diagonal behind the eye; in some individuals postocular ridges present, and dorsolateral folds moderately developed from posterior edge of scapula to sacrum; venter areolate to coarsely areolate, discoidal fold present anterior to the groin; (2) tympanic membrane differentiated from surrounding skin, tympanic annulus visible beneath the skin, rounded, slightly concealed beneath supratympanic fold, tympanum diameter varies from 60% to 90% of the eye diameter; (3) snout short, acutely rounded in dorsal view, rounded in lateral view, with a small rostral papillae at snout tip; (4) upper eyelid tuberculate, with two to three enlarged conical tubercles; upper eyelid wider than interorbital distance; cranial crests absent; (5) dentigerous process of vomer absent; choana small, oval in outline, not covered by palatal floor of maxilla; (6) males lack vocal slits, and lack nuptial pads; (7) Finger I shorter than Finger II; disc narrow equal to or slightly wider than disc on Finger II; (8) fingers with narrow laterals fringes; palmar tubercle is bifid; thenar tubercle barely visible, slightly smaller than palmar tubercle; supernumerary tubercles low and not prominent, distributed on all fleshy parts of palm; hyperdistal subarticular tubercles not prominent, and basal subarticular tubercles rounded; (9) forearms with three small ulnar elongated tubercles; (10) heel bearing few low conical tubercles with an enlarged conical tubercle; outer edge of tarsus bearing three small subconical tubercles, inner tarsal fold present; (11) two metatarsal tubercles, inner oval or rounded, 1.3x–1.5x size of subconical or oval outer tubercle; (12) toes with narrow lateral fringes; supernumerary plantar tubercles numerous, subarticular tubercles defined; Toe V shorter than Toe III, extending between medial and distal subarticular tubercles of Toe IV; (13) in life dorsal coloration grey brown to pearl beige with olive green or orange brown irregular marks, limbs with grey brown to bottle green, and pale brown or chocolate brown transverse bars; ventral coloration green grey to yellow grey, with Khaki grey or olive yellow blotching; iris agate grey, olive green or olive yellow, with an olive drab to black median stripe; (14) SVL males 11.6–16.2 mm (mean 13.43 ± 1.14 mm, *n* = 32); females 19.9–21.2 mm (mean 20.62 ± 0.50 mm, *n* = 5).

**Comparisons with other species (Fig. S1, Table S1).** According to Franco-Mena et al. (2023), *Pristimantis cantrix* (sp. 14) is closely related to *Pristimantis munozi* (Rojas-Runjaic, Delgado & Guayasamin, 2014), *Pristimantis ocreatus* (Lynch, 1981), and *Pristimantis marquezi* (sp. 15); therefore, comparisons of morphological characteristics with these species are presented below. These four species share similar features such as dorsal texture of skin (finely areolate, tuberculated, or rough to slightly smooth), eyelid tuberculated, hyperdistal subarticular tubercles (round and bulky), discoidal fold (present), cranial crests (absent), relative lengths of fingers (Finger I shorter than Finger II), and webbing (absent).

However, other characteristics show its singularities ventral skin is similar in *P. cantrix*, *P, marquezi*, and *P. ocreatus* with a texture ranging from slightly to heavily aerolated; *P. munozi* also shares this feature but may exhibit a smooth to slightly areolate texture on the throat. Postrictal tubercles in *P. cantrix* are conical and small to moderately sized; while in *P. marquezi* are conical and long; *P. ocreatus* has a non-conical postrictal tubercle. Presence of ulnar tubercles in *P. cantrix*: three conical tubercles, *P. marquezi:* three conical tubercles in lateral folds, and *P. ocreatus* has no distinctive tubercles. Inner tarsal fold present in *P. cantrix*, *P. munozi*, *P. ocreatus*, and *P. marquezi*. Snout shape in *P. cantrix* is acutely rounded in dorsal view, rounded in lateral view, with a small rostral papilla at snout tip; in *P. munozi* is short, acuminate in dorsal view, rounded in profile; in *P. marquezi* subacuminate or acutely rounded in dorsal view, rounded in lateral view, with small rostral papilla at snout tip; in *P. ocreatus* is very short, round in dorsal and lateral profile. Canthus rostralis in *P. cantrix*, *P. munozi*, and *P. marquezi* is weakly concave; in *P. ocreatus* is rounded to obtuse. Lips not flared in *P. cantrix*, *P. ocreatus*, and *P. marquezi*. Tympanum in *P. cantrix* is evident, round (3/5–9/10 of eye diameter), posterodorsally slightly concealed beneath supratympanic fold; in *P. munozi* is small, but well-defined (1/2–2/5 of eye diameter), slightly higher than long, posterodorsally concealed beneath skin; in *P. ocreatus* is concealed beneath skin; and in *P. marquezi* is evident, round (3/5–4/5 of eye diameter), posterodorsal slightly concealed beneath supratympanic fold. Dentigerous processes of vomers in *P. cantrix* and *P. ocreatus* are absent; in *P. munozi* are low, triangular, slightly smaller than choanae, arranged in V-shaped, posterior and medial to choanae, each bearing one to four teeth; in *P. marquezi* are low, obliquely angled posteriomedially. Vocal slits in *P. cantrix* absent, in *P. munozi* prominent, in *P. ocreatus* short, and in *P. marquezi* not visible. Vocal sac in *P. cantrix* and *P. marquezi* subgular, and in *P. ocreatus* internal. Nuptial pads in *P. cantrix*, *P. munozi*, and *P. marquezi* absent.

Dorsolateral folds in *P. cantrix*, *P. munozi*, *P. ocreatus*, and *P. marquezi* are present and may be from posterior edge of scapula to sacrum, but in *P. cantrix* could be from posterior edge of eyelid to sacrum also. Lateral folds are occasionally present in some individuals of *P. cantrix* as rows of low ridges and tubercules, and in *P. marquezi* as rows of low conical tubercles. Interocular fold absent in *P. munozi*, *P. ocreatus*, and *P. marquezi*; but *P. cantrix* have a row of low tubercles. Supratympanic fold present in *P. cantrix* and *P. marquezi*; not prominent in *P. ocreatus*. Paravertebral folds are mostly absent and, in some individuals, present as low ridges in *P. cantrix*, and present as low irregular ridges in *P. marquezi* and *P. ocreatus*. Middorsal fold is mostly absent or sometimes present as a row of low tubercles from frontoparietal to sacrum in *P. cantrix*, absent in *P. marquezi*. Occipital-scapular folds present in *P. cantrix* and *P. marquezi* as sinusoidal, W, or )( shaped scapular ridges, and in some individual’s postocular ridges as part of dorsolateral folds; *P. munozi* also has dorsolateral folds. Thoracic fold present in *P. cantrix*, and *P. marquezi*. Heels in *P. cantrix, P. munozi* and *P. marquezi* are described as tuberculated with few and low tubercles; *P. ocreatus* doesn’t have enlarged or distinctive heel tubercles. Tarsal tubercles in *P. cantrix*, *P. marquezi* and *P. munozi* are present, but with its own singularities, such as, *P. munozi* have four to five-fold-like tubercles, *P. cantrix* three small conical tubercles, *P. marquezi* three small triangular or fold-like tubercles; in contrast, *P. ocreatus* has no enlarged or distinctive tarsal tubercles. In terms of plantar tubercles, *P. munozi*, *P. cantrix*, *P. marquezi*, and *P. ocreatus* show two metatarsal tubercles, inner oval and larger than outer. *P. cantrix* shows the shorter difference between metatarsal tubercles size, with inner tubercle 1.3–1.5 times outer tubercle, followed by *P. marquezi* with 1.5–1.8 times bigger, *P. munozi* about two times bigger, and *P. ocreatus* two to three times size of outer tubercle.

The lateral fringes of fingers in *P. cantrix* and *P. marquezi* are narrow, in *P. munozi* preaxial keel on Finger II–Finger III, and absent in *P. ocreatus*. Discs of fingers in *P. cantrix* are narrow, rounded, slightly elongated acuminate or expanded; in *P. munozi* are narrow and rounded; in *P. ocreatus* as long as wide on Finger III and Finger IV; and in *P. marquezi* Finger I and Finger II discs are narrow and rounded, while Finger III and Finger IV are slightly expanded and elliptical. Palmar tubercle is bifid in *P. cantrix*, *P. marquezi*, and *P. ocreatus*, but *P. cantrix* may present single round or oval tubercle, whereas *P. marquezi* only oval tubercle, or divided ovals. Thenar tubercle is almost as large as palmar tubercle; in *P. marquezi* thenar tubercle may be barely visible, and in *P. cantrix* palmar tubercles may be barely visible. Hyperdistal subarticular tubercles not prominent, and basal subarticular tubercles rounded in *P. cantrix*. Supernumerary tubercles in *P. cantrix* and *P. marquezi* are round, small, and plump, whereas in *P. ocreatus* are indistinct; Toe V shorter than Toe III, extending to the medial subarticular tubercles of Toe IV in *P. cantrix* and *P. marquezi*; whereas *P. munozi* has Toe V slightly longer than Toe III, and not extending to the proximal edge of the distal subarticular tubercle of Toe IV. The lateral fringes of toes in *P. cantrix* and *P. marquezi* are narrow, whereas in *P. munozi* and *P. ocreatus* are absent. Discs on toes in *P. cantrix* and *P. marquezi* are rounded, narrow, slightly elongated acuminate, while in *P. munozi* are slightly expanded.

**Description of the holotype (Fig. 3).** Adult male (ZSFQ 4466), head longer than wide, length and width 40.9% SVL; snout broadly rounded in dorsal and lateral view. Eye nostril distance 9.7% SVL; canthus rostralis weakly concave and loreal region slightly concave; nostril slightly protuberant oriented laterally, in lateral view; interorbital area flat, wider than the upper eyelid, upper eyelid represents 82.3% interorbital distance; cranial crests absent; body with long dorsolateral folds well developed; upper eyelid with two subconical tubercles (rounded in preservative); canthus rostralis and loreal region lack tubercles; tympanic membrane differentiated from surrounding skin, tympanic annulus visible beneath the skin, rounded, laterally oriented, tympanum diameter 57.8% of the eye diameter; small choanae, rounded in outline; dentigerous process of vomer absent, oval tongue longer than wide. Skin on dorsum finely shagreen with rounded tubercles widespread at dorsolateral folds notched posteriorly; venter areolate with some pustules, discoidal fold anterior to the level of the groin; anal ornamentation absent, with few rounded tubercles; forearms slender. Fingers with fine lateral fringes, palmar tubercle oval smaller than thenar; subarticular tubercles rounded and defined, with supernumerary tubercles at the base of each digit; discs narrow, narrow lateral fringes; all fingers have digital pads defined by circumferential grooves; hyperdistal subarticular tubercles not prominent, and basal subarticular tubercles rounded. Hindlimbs wide, tibia length 47.22% SVL, two subconical tubercles on the heel, a row of subconical tubercles on the outer edge of tarsus; inner tarsal fold present; Toes without lateral fringes and digital webbing, digital pads of the toes small, rounded, narrow lateral fringes; rounded subarticular tubercles, supernumerary tubercles weakly defined rounded; inner metatarsal tubercles present; Toe III longer than Toe V, Toe III reaching to the base of distal subarticular tubercle of Toe IV.

**Measurements (in mm) of the holotype (ZSFQ 4466).** SVL = 14.4, HL = 5.9, HW = 5.9, IOD = 1.7, UEW = 1.4, EL = 1.9, END = 1.4, TY = 1.1, IND = 1.8, UAL = 3.1, FLL = 3.4, HAL = 3.6, Finger I = 1.9, Finger II = 2.1, Finger III DW = 0.5, THL = 6.1, TL = 6.8, FL = 6.2, and Toe IV DW = 0.5.

**Color of holotype in life (Fig. 3).** Dorsal surfaces light to dark brown, dorsolateral lines dark brown, laterally lighter with light brown to light green shades and forelimbs are lead colored with small white spicules. The dorsal surfaces of the legs are light brown with contrasting dark brown bars. Its belly is dark brown; the gular region has a little creamy brown tint. Groin in females present orange to red spots. The iris is pale greenish copper yellow with a dark red mid horizontal bar and an orange copper ring around the pupil.

**Color of holotype in ethanol (Fig. 3).** Dorsal surfaces present a dark brown color with pale brown spots with a middorsal band brown formed by pale brown flanks with small dark markings. Upper and lower lip with dark brown bars, dark brown supratympanic line. Dorsum of legs brown with dark brown bars. Cloacal region dark brown. Groin cream brown, upper and lower surface of thighs pale brown. The gular region is cream brown with dark markings in the center. Belly pale brown. Most of the ventral surface is dark brown with tubercles not prominent, arms and legs ventrally light brown. Palmar surfaces cream brown and plantar surfaces dark brown, tubercles on palms and feet unpigmented.

**Figure 4:**
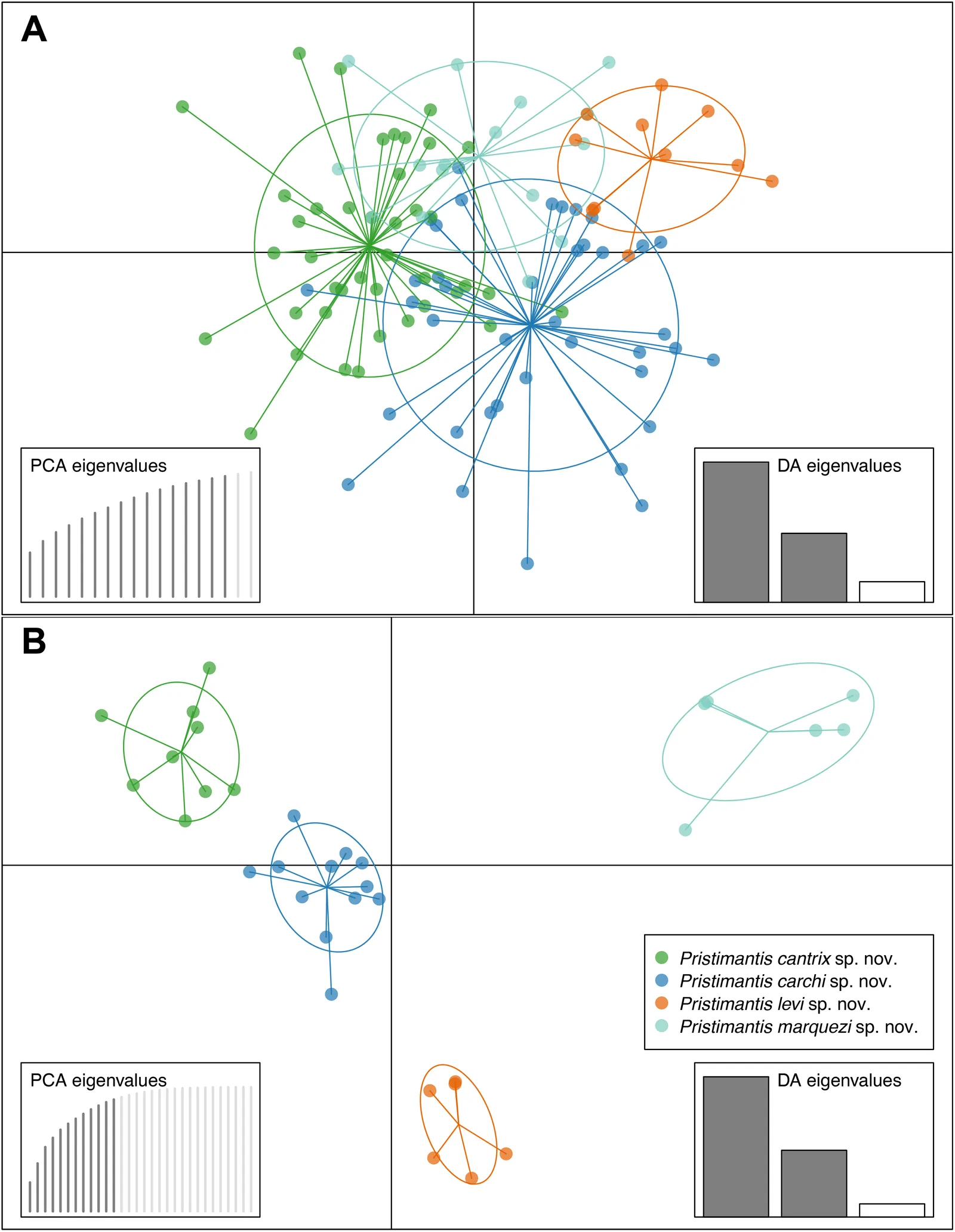
Scatterplot of the discriminant analyzes (DAPC). (A) On the morphometric dataset of *Pristimantis cantrix* sp. nov., *Pristimantis carchi* sp. nov., *Pristimantis levi* sp. nov., and *Pristimantis marquezi* sp. nov., 16 first PCs, 96% retained variance. (B) On the acoustic dataset, 12 first PCs, 90% retained variance.

**Figure 5:**
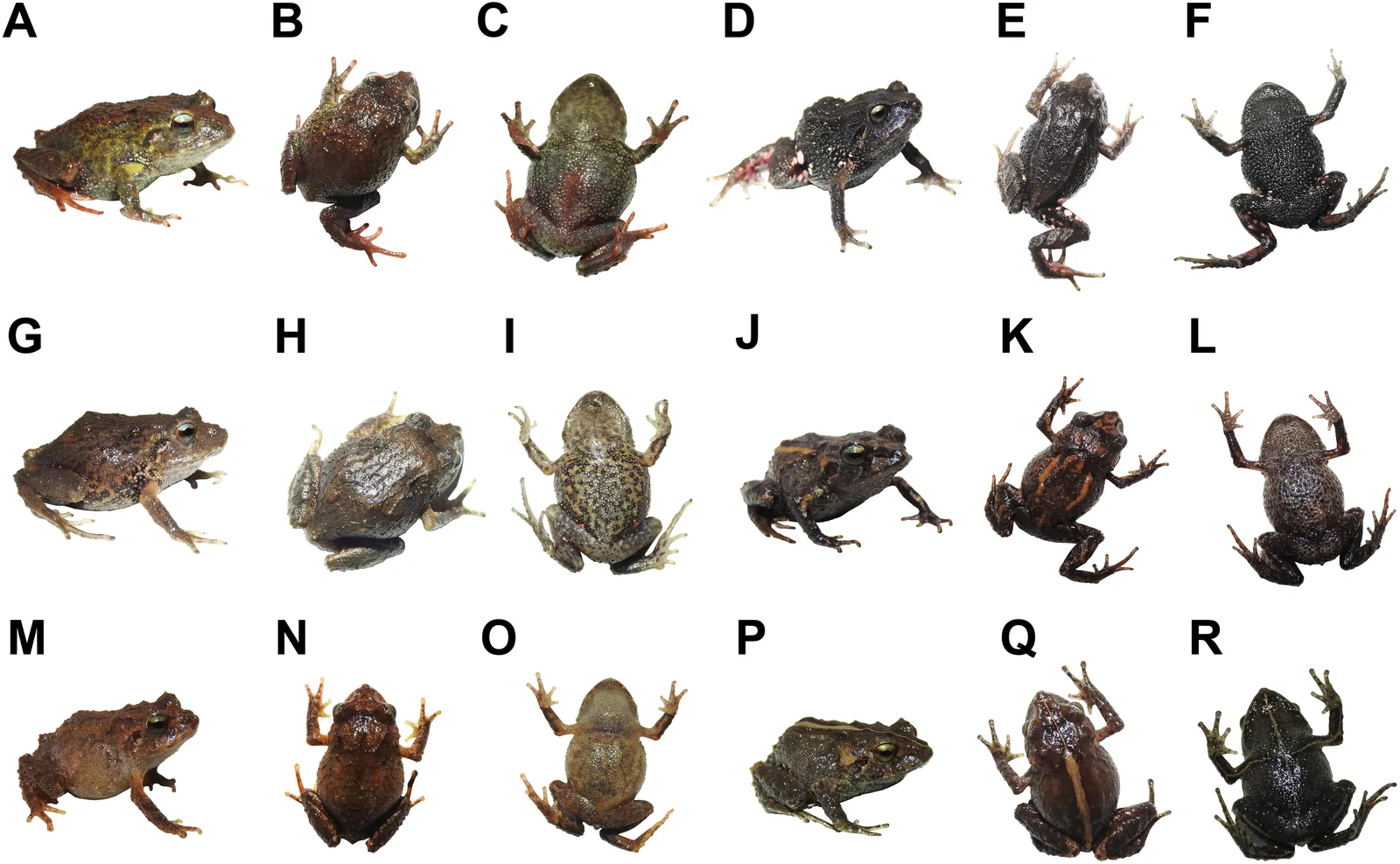
Color variation in life of *Pristimantis cantrix* sp. nov. Photographs in lateral, dorsal, and ventral view. (A–C) ZSFQ 4551 female, (D–F) ZSFQ 4572 female, (G–I) ZSFQ 4555 female, (J–L) ZSFQ 4467 male, (M–O) ZSFQ 4514 male, (P–R) ZSFQ 4521 male. Photographs by Diego Batallas.

**Variation.** Morphometric and acoustic variation is shown in Appendix S1 and Fig. 4. *Pristimantis cantrix* sp. nov. shows variation in color (Figs. 5, S1). Interorbital bar, if present, may show coloration from grey brown to olive drab. Canthal stripe displays different tones of brown, such as black brown, chocolate brown, black, olive drab, brown grey, or black red. Labial bars have tones of brown such as olive drab, black brown, chocolate brown, or black, and in some individuals, lips have platinum grey interspaces. Dorsal coloration varies from uniform patterns of brown, with or without irregular marks of olive green or orange brown, and in some individuals longitudinal stripes of terra brown, ochre yellow, grey beige or olive drab. Axilla and groin exhibit green grey tones on the background, and in some individuals cream spots. Transverse bars are evident with pale brown or chocolate brown, but inconspicuous in other individuals. Venter shows green grey to yellow grey tones, and brown grey to platinum grey blotching, but in one specimen (ZSFQ 4521) brown grey on longitudinal and transversal stripe in the middle of the venter and chest.

**Figure 6:**
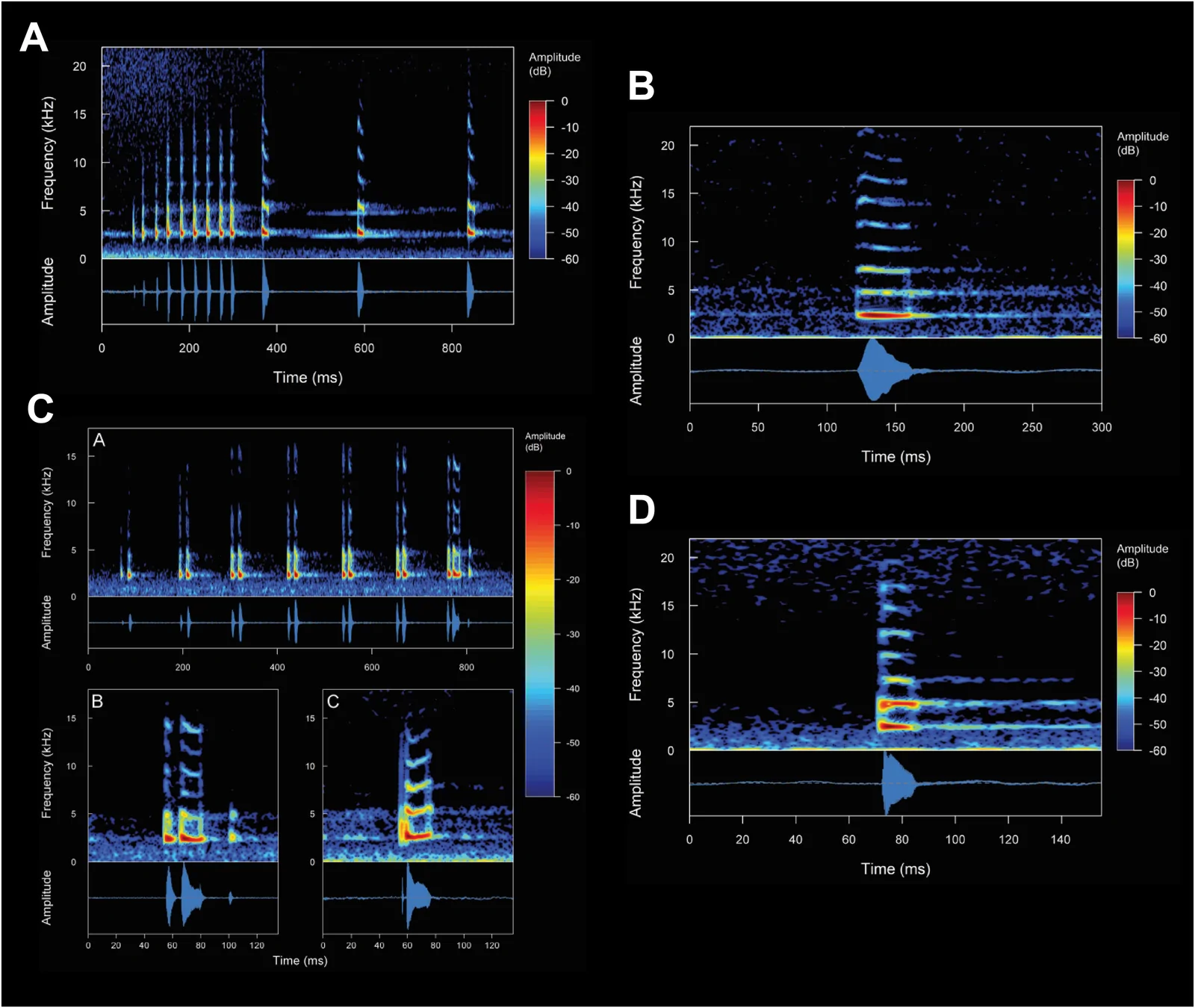
Oscillograms and spectrograms of the advertisement calls of the new species of *Pristimantis myersi* group. (A) *Pristimantis cantrix* sp. nov. (ZSFQ 4512, SVL 13.35 mm, 9.9 °C air temperature, 76% relative humidity), (B) *Pristimantis carchi* sp. nov. (ZSFQ 4565, SVL 18.86 mm, 11.5 °C air temperature, 89% relative humidity), (C) *Pristimantis levi* sp. nov. (A complete advertisement call, B detail of a pulsed note with downward frequency modulation (A, B) DHMECN 13334, SVL 17.1 mm, 8.3 °C air temperature, 78% relative humidity, and (C) detail of a pulsed note with upward frequency modulation (ZSFQ 4500, SVL 18.27 mm, 11.8 °C air temperature, 93% relative humidity), and (D) *Pristimantis marquezi* sp. nov. (ZSFQ 4427, SVL 15.28 mm, 7.9 °C air temperature, 82% relative humidity). Modified from Batallas, Márquez & Guayasamin (2025). Prepared by Diego Batallas.

**Calls (Fig. 6A).** The description of the call of *Pristimantis cantrix* is published in Batallas, Márquez & Guayasamin (2025) (see *Pristimantis* sp. 3). In this work the principal acoustic information is provided in Table 1.

**Table 1:** Summary of acoustic status (mean and range) of calls provided in Batallas, Márquez & Guayasamin (2025) of the new species in this study.

Species	Sounds	Calls (*n*)	Mean	Mean intervals	Notes	First note	Mean intervals	Mean dominant frequency
*Pristimantis cantrix* sp. nov. (*Pristimantis* sp. 3)	Sounds are like a constant metallic tapping that onomatopoeically resembles a “tic”.	295	630.17 ± 281.68 ms (range 207–1,294 ms)	3.89 ± 1.2 s (range 2.29–12.92 s)	mean of 3.01 ± 1.11 notes (range 1–5 notes)	239.18 ± 79.5 ms (range 104–490 s)	39.33 ± 14.21 ms (range 4–93 ms)	2.55 ± 0.09 kHz (range 2.33–2.84 kHz).
*Pristimantis carchi* sp. nov. (*Pristimantis* sp. 2)	Sounds are like a kind of metallic tapping that onomatopoeically resembles a “tic”.	25	225.79 ± 263.9 ms (range 8–1,042 ms)	3.8 ± 1.71 s (range 2.15–21.45 s)	1.6 ± 0.63 notes (range 1–3 notes)	14.01 ± 4.89 ms (range 9–39 ms)	407.51 ± 91.52 ms (range 302–798 ms)	2.63 ± 0.13 kHz (range 2.33–2.84 kHz)
*Pristimantis levi* sp. nov. (*Pristimantis* sp. 5)	na	127	357–827 s (range 502.45 ± 123.07 s)	22.26 ± 13.55 s (range 2.89–57.66 s)	6.85 ± 0.96 notes (range 5–9 notes)	20.88 ± 7.29 ms (range 2–48 ms)	61.34 ± 12.7 ms (range 40–108 ms)	2.48 ± 0.14 kHz (range 2.15–2.84 kHz
*Pristimantis marquezi* sp. nov. (*Pristimantis* sp. 4)	Sounds are like a kind of metallic tapping that onomatopoeically resembles a “tic”.	181	149.16 ± 251.15 ms (range 9–903 ms)	5.21 ± 0.87 s (range 3.85–9.15 s)	1.24 ± 0.43 notes (range 1–2 notes)	11.88 ± 1.07 ms (range 10–13 ms)	567.93 ± 94.17 ms (range 467–877 ms)	2.57 ± 0.08 kHz (range 2.33–2.76 kHz)

**Figure 7:**
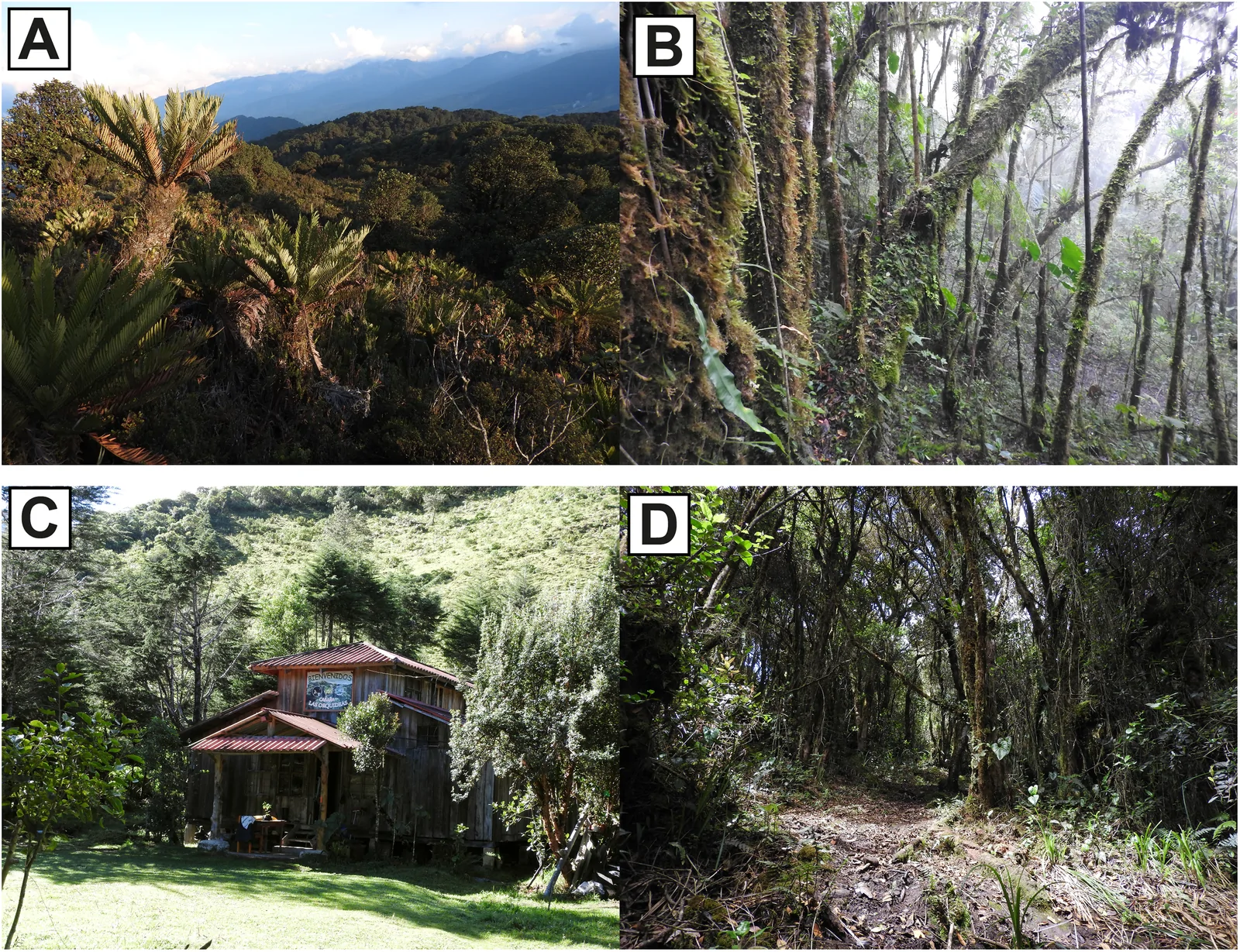
Characteristic habitats of the type localities of the new species of the *Pristimantis myersi* group, described from Carchi province in northern Ecuador. (A) Cordillera de la virgen negra, *Pristimantis cantrix* sp. nov; (B) La Changadera-Morán, *Pristimantis carchi* sp. nov; (C) Cabaña las orquídeas-Morán, *Pristimantis levi* sp. nov; (D) San francisco de Pioter, *Pristimantis marquezi* sp. nov. Photographs by Diego Batallas.

**Distribution and natural history (Figs. 1, 7).** During this study, *Pristimantis cantrix* sp. nov. was collected at three high-Andean localities in the province of Carchi, northern Ecuador: Virgen Negra, Cerro La Bretaña, and Loma La Esperanza, ranging from 3,019 to 3,612 m. These sites correspond to the Northeastern Andean High Mountain Evergreen Forest, which is characterized by trees reaching up to 15 m in height (*e.g*., *Clusia)*, dense undergrowth, tree ferns, and abundant epiphytes; and the Caulescent Rosette and Páramo Grassland (frailejones), dominated by herbaceous vegetation including pajonal, cushions, and *Espeletia pycnophylla* (Ministerio del Ambiente del Ecuador (MAE), 2013).

This species exhibits both diurnal and nocturnal habits and has been observed perched or partially buried in leaf litter, near adventitious roots, fallen logs, and at the base or inside trees, ferns, and frailejones of the genus *Espeletia* (Batallas, Márquez & Guayasamin, 2025). *Pristimantis cantrix* sp. nov. is sympatric and syntopic with *P. festae* and *P. carchi* sp. nov., especially in transitional high Andean habitats where these species coexist. As a result, its identification in the field is difficult during initial surveys, particularly at night. Reliable differentiation depends mainly on morphometric and phenotypic traits in adult females, while in males, the most distinctive feature is their characteristic advertisement call (Batallas, Márquez & Guayasamin, 2025).

**Evolutionary relationships (Fig. 2).** In the *Pristimantis myersi* species group, it is closely related to *P*. *marquezi* sp. nov., *P. munozi*, and *P. ocreatus* (Franco-Mena et al., 2023).

**Etymology.** The specific name *cantrix* is derived from the latin word for “singer” or “chantress.” This term refers to the distinctive advertisement call of males, which serves as a key trait for species identification in the field (see Batallas, Márquez & Guayasamin (2025) for acoustic characteristics, where the species is referred to as *Pristimantis* sp. 3).

**Conservation status.** *Pristimantis cantrix* sp. nov. is known from only three localities in the Carchi province, an area that has suffered deforestation and fragmentation because of agriculture and cattle farming (Individuals found during this study were located in relatively well-preserved patches of forest, surrounded by fragmentation). The current estimated extent of occurrence for the species is <100 km^2^. Therefore, following IUCN criteria to assess the current extinction risk of the species (Gärdenfors et al., 2001), we propose that *P. cantrix* sp. nov. should be considered as Critically Endangered (CR) B1 a, b (i, iii).

***Pristimantis carchi* sp. nov**

*Pristimantis* sp. 12, Franco-Mena et al., 2023

*Pristimantis* sp. 2 Batallas, Márquez & Guayasamin, 2025

**LSID.** urn:lsid:zoobank.org:act:3148008B-EFE2-4146-B5A6-F1702444135C

**Proposed standard English name:** Carchi Rain Frog

**Proposed standard Spanish name:** Cutín de Carchi

**Figure 8:**
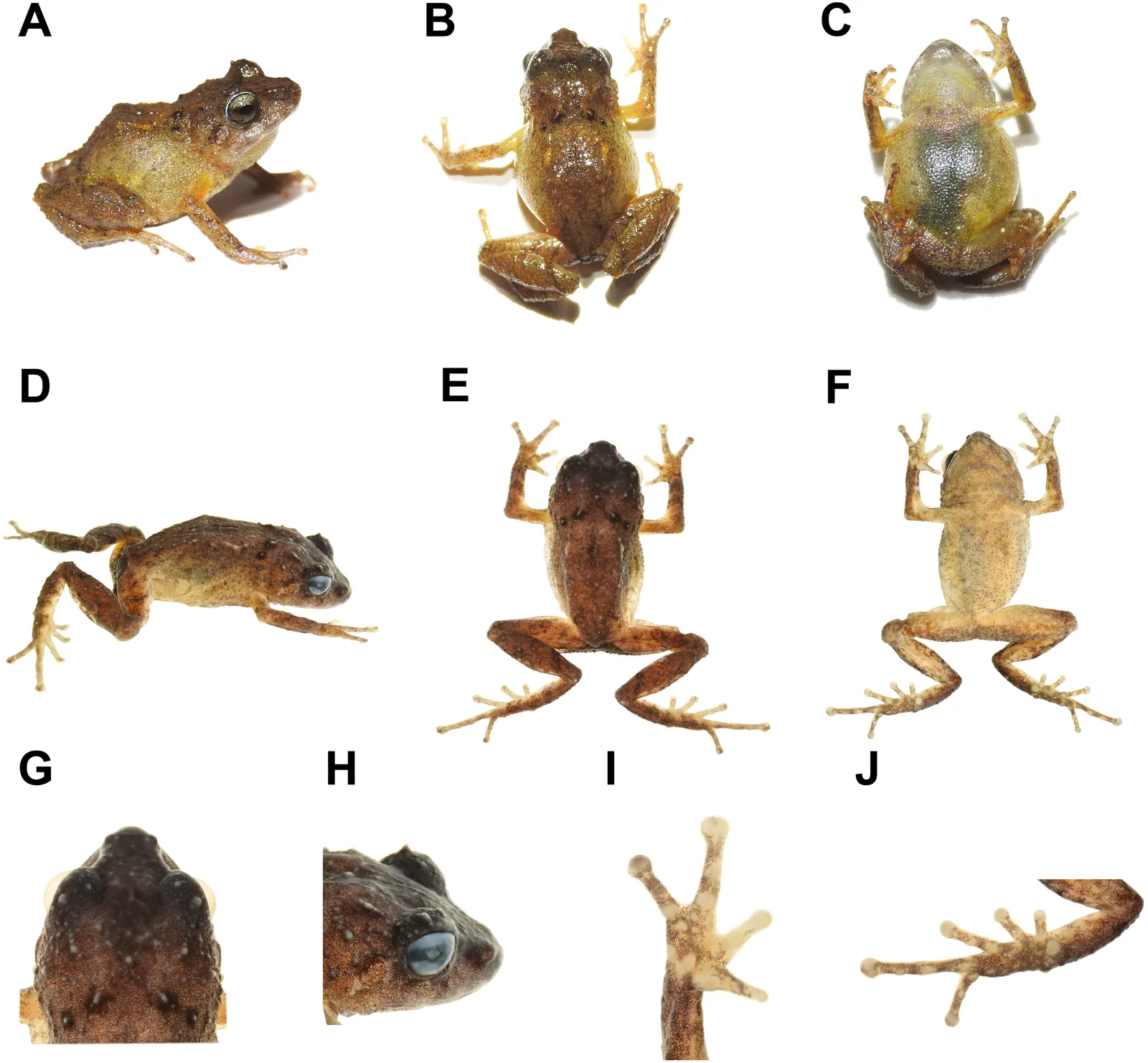
Holotype of *Pristimantis carchi* sp. nov. (ZSFQ 4502), adult male, SVL = 17.1 mm. (A–C) Live and (D–F) preserved photographs in lateral, dorsal and ventral view; (G) head in dorsal and (H) lateral view, (I) palmar surface, and (J) plantar surface. Photographs by Diego Batallas, Malki Bustos, and Daniela Franco-Mena.

**Holotype (Fig. 8).** ZSFQ 4502, adult male, collected on 25 March 2020 by Diego Batallas and Carlos Castro at Morán (0.76925 N, 78.042639 W; 3,076 m), Libertad, Espejo, Carchi province, Ecuador.

**Paratypes.** A total of 36 specimens, including adult males (24) and females (3), were collected between January 2020 and February 2021 across five localities in Carchi province, Ecuador. ZSFQ 4505 (male), with same data as the holotype. ZSFQ 4484 (male), collected on 18 March 2020 by Diego Batallas and Carlos Castro at Morán (0.764944 N, 78.043999 W; 2,950 m), Libertad, Espejo, Carchi province, Ecuador. ZSFQ 4533, 4534 (males), collected on 10 February 2021; ZSFQ 4535, 4536, 4537, 4538 (males), collected on 11 February 2021; ZSFQ 4539, 4540 (males), ZSFQ 4541 (female), collected on 13 February 2021 by Diego Batallas and Carlos Castro at Morán (0.774444 N, 78.041528 W; 3,056 m), Libertad, Espejo, Carchi province, Ecuador. ZSFQ 4570, 4572 (males), ZSFQ 4573 (female), collected on 28 February 2020 by Diego Batallas, Gabriela Olmedo and Jaime Rosero at Cerro La Bretaña (0.566694 N, 77.718278 W; 3,150 m), Fernández Salvador, Montúfar, Carchi province, Ecuador. ZSFQ 4565, 4576, 4577, 4578 (males), ZSFQ 4579 (female), collected on 29 February 2020 by Diego Batallas, Gabriela Olmedo and Jaime Rosero at Cerro La Bretaña (0.567000 N, 77.713250 W; 3,255 m), Fernández Salvador, Montúfar, Carchi province, Ecuador. ZSFQ 4446 (male), collected on 22 January 2020; ZSFQ 4447 (male), collected on 23 January 2020; ZSFQ 4448, 4449, 4450 (males), collected on 24 January 2020; ZSFQ 4451, 4452, 4453 (males), collected on 25 January 2020 by Diego Batallas, Fernando Paguay and Valeria Ayo at Camino Tufiño–Maldonado (0.802778 N, 78.004667 W; 3,362 m), Tufiño, Tulcán, Carchi province, Ecuador.

**Diagnosis.** *Pristimantis carchi* sp. nov. can be distinguished from other *Pristimantis* by the following character combination: (1) skin on dorsum finely tuberculate with scattered larger tubercles; straight, sinusoidal, W, ∨, or )( shaped scapular ridges; from posterior edge of eyelid to scapula, dorsolateral folds weakly devoloped; venter weakly areolate to areolate, discoidal fold present at the level of the groin; (2) tympanic membrane differentiated from surrounding skin, tympanic annulus visible beneath the skin, rounded, posterodorsal slightly concealed beneath supratympanic fold, tympanum diameter varies from 60% to 80% of the eye diameter; (3) snout short, acutely rounded in dorsal view, rounded in lateral view, in some truncate at snout tip; (4) upper eyelid tuberculate, with two enlarged conical tubercles; upper eyelid wider than interorbital distance; cranial crests absent; (5) dentigerous process of vomer low, obliquely angled posteromedial; (6) males lack vocal slits, and subgular vocal sac, nuptial pads; (7) Finger I shorter than Finger II; discs narrow, rounded and some individuals truncate; (8) fingers with narrow laterals fringes; palmar tubercle single or divided, if single oval or slightly bifid distally; thenar tubercle oval or irregular, and barely visible, equal than palmar tubercle; supernumerary tubercles, distributed on the basal parts of palm; hyperdistal subarticular tubercles, and basal subarticular tubercles rounded; (9) forearms with small and rounded ulnar tubercles; (10) heel bearing few low round tubercles with an enlarged conical tubercle; outer edge of tarsus bearing three small triangle or fold-like tubercles, inner tarsal fold present; (11) two metatarsal tubercles, inner oval 1.4x–2.5x size of subconical or round outer tubercle; (12) toes with narrow lateral fringes; supernumerary plantar flattened tubercles; Toe V shorter than Toe III, extending between medial and distal subarticular tubercles of Toe IV; (13) in life, dorsal coloration pale brown to black with chocolate brown to olive yellow irregular marks, limbs with grey brown to orange brown, and fawn brown transverse bars; ventral coloration olive yellow to terra brown; iris pebble grey, pale green, or mouse grey, with a black brown or olive drab median stripe; (14) SVL males 12.4–18.9 mm (mean 15.14 ± 1.34 mm, *n* = 24); females 19.9–21.5 mm (mean 19.84 ± 1.79 mm, *n* = 3).

**Comparisons with other species (Fig. S2, Table S1).** According to Franco-Mena et al. (2023), *Pristimantis carchi* is closely related to *Pristimantis leoni* (Lynch, 1976b); therefore, comparisons of morphological characteristics with this species are presented below. Both species share similar features such as ventral texture of skin (areolate), inner tarsal fold (present), dentigerous processes of vomers (low and obliquely angled posteromedially), vocal sac in males (present), relative lengths of fingers (Finger I shorter than Finger II), lateral fringes of toes (present), and webbing (absent).

However, other characteristics show its singularities, the dorsal texture of skin in *P. carchi* is finely tuberculate with scattered larger tubercles, while in *P. leoni* is shagreened with numerous short ridges. Eyelids are tuberculate in both, but in *P. carchi* has two enlarged conical tubercles, and in *P. leoni* one or more enlarged conical tubercles. Dorsolateral folds present in *P. carchi* as postocular folds from posterior edge of eyelid to sacrum, or from posterior edge of scapula to sacrum; whereas absent in *P. leoni*. Supratympanic fold present in *P. carchi*, in *P. leoni* if present is obscured by tubercles. Discoidal fold present in *P. carchi*, absent in *P. leoni*. The snout shape in *P. carchi* is acutely rounded in dorsal view, rounded in lateral view, and in some individuals truncate at snout tip; whereas in *P. leoni* is short, subacuminated in dorsal view (tip feebly pointed), and semitruncate in lateral view. Canthus rostralis is similar in *P. carchi* and *P. leoni*, both could be sharp and concave, but some individuals of *P. carchi* show straight canthus rostralis. Cranial crests absent in *P. carchi* and *P. leoni*. Lips not flared in *P. carchi* and *P. leoni*. Tympanum in *P. carchi* is evident, round (3/5–4/5 of eye diameter), posterodorsal slightly concealed beneath supratympanic fold, larger than *P. leoni*, which is higher than long (1/3–2/5 of eye diameter), and partially concealed beneath skin. Vocal slits not visible in *P. carchi*, present in *P. leoni*.

The lateral fringes of fingers are narrow in *P. carchi*, absent in *P. leoni*. Discs of fingers in *P. carchi* are narrow, rounded, or truncate, whereas in *P. leoni* discs are present in Finger II, Finger III, and Finger IV, and tips of digits almost round. Palmar tubercle in *P. carchi* could be single or divided, if single oval or slightly bifid distally, if divided in two or three oval segments, thenar tubercle oval or irregular, and barely visible; in *P. leoni* is bifid, larger than oval thenar tubercle. Hyperdistal subarticular tubercles in *P. carchi* are round and bulky, while in *P. leoni* are wider than long and flat. Heels are tuberculate in *P. carchi* and *P. leoni*, but with one enlarged conical tubercle in *P. carchi*, not elongated or prominent tubercle in *P. leoni*. Tarsal tubercles in *P. carchi* are three small triangular or fold-like outer tubercles, while one to three small, conical tubercles in *P. leoni*. Plantar tubercles in *P. carchi* are two metatarsal bulky tubercles, inner tubercle is oval 1.4x–2.5x size of subconical or round outer tubercle, whereas in *P. leoni* are two metatarsal tubercles, inner tubercle a little longer than wide, not compressed, and outer tubercle round, subconical, one-half size of inner.

**Description of the holotype (Fig. 8).** Adult male (ZSFQ 4502), head wide than longer, length 38.5% SVL, width 39.7% SVL; snout subacuminate in dorsal and rounded in lateral view. Eye nostril distance 8.18% SVL; canthus rostralis concave and loreal region slightly concave; nostrils slightly protuberant, oriented laterally, interorbital area flat, wider than upper eyelid; interorbital area flat, wider than the upper eyelid, upper eyelid represents 89.4% interorbital distance; cranial crests absent; occipital fold defined by the presence of two pairs of prominent subconic tubercles on the occipital and scapular regions; row of small rounded tubercles from the tip of the snout to the interorbital region; upper eyelid with small tubercles and two prominent subconical tubercles (rounded in preservative); rounded tubercles scattered on the canthus rostralis; tympanic membrane differentiated from surrounding skin, tympanic annulus visible, rounded, laterally oriented, tympanum diameter 56.5% of the eye diameter; small choanae, rounded in outline; dentigerous process of vomer present, triangular in outline, minute and embedded in roof of mouth, oval tongue. Skin on dorsum shagreen with rounded tubercles widespread and dermal ridges; venter areolate with some scattered warts, discoidal fold at the level of the groin; anal ornamentation present, with rounded tubercles; forearms slender with subconical ulnar tubercles reduced and a row of subconical tubercles along the anterior edge (reduced or less evident in preservation effects); Fingers with fine lateral fringes, palmar tubercle oval the same size and shape than thenar; subarticular tubercles rounded and defined, with supernumerary tubercles at the base of each digit; digital pads barely expanded; hyperdistal subarticular tubercles absent; palmar tubercle divided; thenar tubercle oval and visible; hyperdistal subarticular tubercles, and basal subarticular tubercles rounded. Hindlimbs slender, tibia length 48.5% SVL, one subconical tubercle on the heel, a row of subconical tubercles on the outer edge of tarsus; inner tarsal fold present; toes with fine lateral fringes, with basal digital webbing between Toe IV and Toe V, digital pads of the toes expanded; low and rounded subarticular tubercles, supernumerary tubercles weakly defined rounded; metatarsal tubercles present, inner oval more bigger than outer tubercle that is rounded; Toe III longer than, reaching the base of distal subarticular tubercle of Toe IV.

**Measurements (in mm) of the holotype (ZSFQ 4502).** SVL = 17.1, HL = 6.6, HW = 6.8, IOD = 1.9, UEW = 1.7, EL = 2.3, END = 1.4, TY = 1.3, IND = 2.0, UAL = 3.6, FLL = 4.8, HAL = 4.8, Finger I = 2.3, Finger II = 2.4, Finger III DW = 0.9, THL = 8.5, TL = 8.3, FL = 8.4, and Toe IV DW = 0.8.

**Color of holotype in life (Fig. 8).** Dorsal surfaces are light to dark brown, laterally lighter with light brown to yellowish shades and small white spicules on forelimbs. The dorsal surfaces of the legs are brown with contrasting dark brown bars. Brown labial bars. Belly yellowish with a few yellow or orange dots or bangs, the gular region has creamy yellow tints. Iris yellowish brown with black reticulations.

**Color of holotype in ethanol (Fig. 8).** Dorsal surfaces are dark brown with pale brown spots, on the flanks pale brown with small dark markings. Upper lip is cream brown, and the upper tympanic line is dark brown. The posterior surface of legs is brown with dark brown bars. Cloacal region dark brown. Groin, upper and lower surface of thighs pale brown. Ventral pattern yellowish cream with flecks evenly distributed on throat, chest, and belly. Arms and legs ventrally yellowish cream with scattered light brown spots. Palmar and plantar surfaces dark brown, tubercles on palms and soles unpigmented.

**Variation.** Morphometric and acoustic variation is shown in Appendix S1 and Fig. 4.

**Figure 9:**
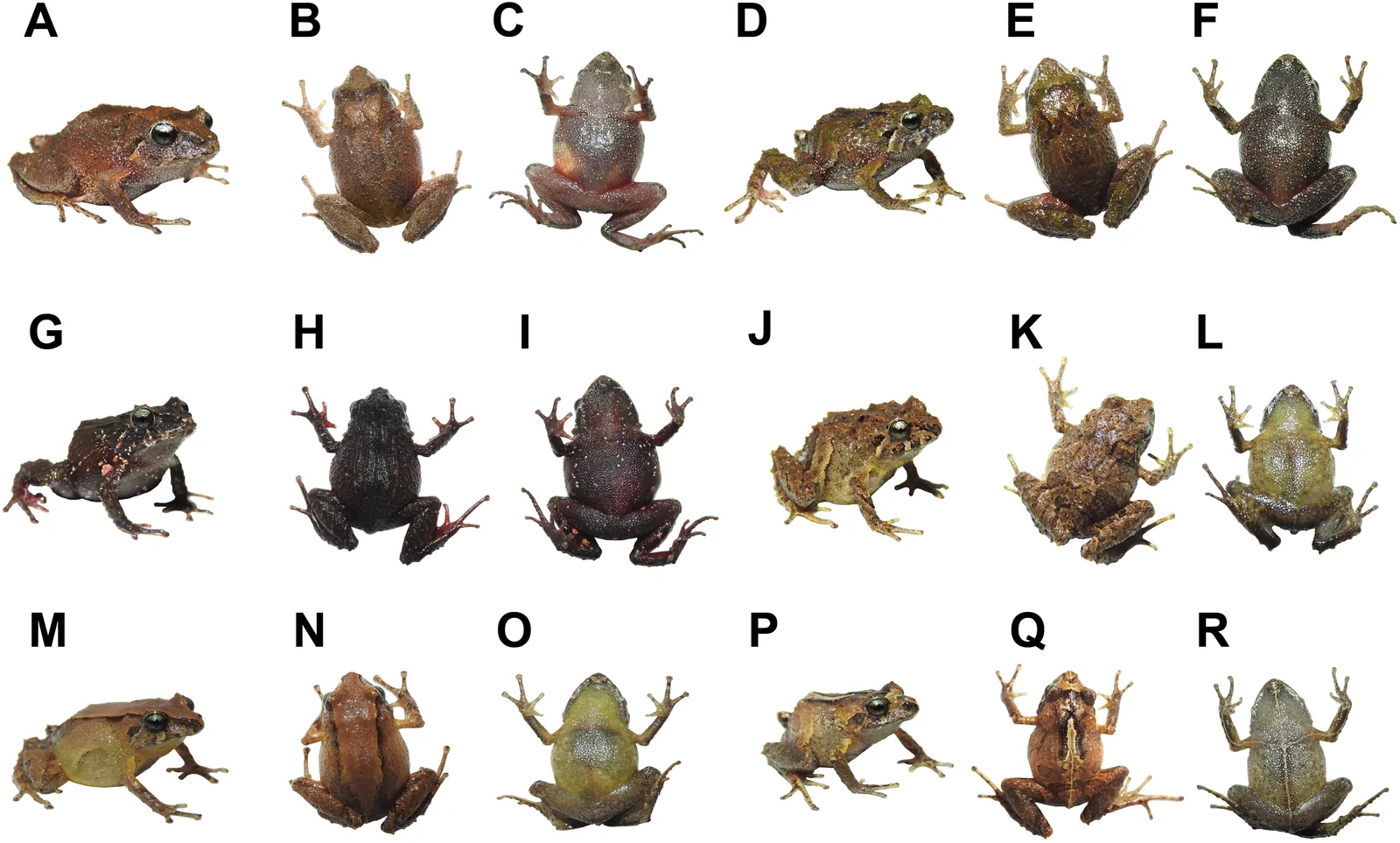
Color variation in life of *Pristimantis carchi* sp. nov. Photographs in lateral, dorsal, and ventral view. (A–C) ZSFQ 4573 female, (D–F) ZSFQ 4579 female, (G–I) ZSFQ 4551 female, (J–L) ZSFQ 4479 male, (M–O) ZSFQ 4477 male, (P–R) ZSFQ 4482 male. Photographs by Diego Batallas.

*Pristimantis carchi* sp. nov. shows variation in color (Figs. 9, S2). Interorbital bar, if present, may show coloration from brown to black grey. Canthal stripe and postorbital stripe display different tones of brown, such as mahogany brown, olive drab, chocolate brown, or umbra grey. Labial bars in olive drab or brown, may be accompanied by beige stripes. Dorsal coloration varies from uniform patterns of brown, with irregular marks chocolate brown, mahogany brown, or olive yellow. Axilla and groin in males exhibit beige tones, in females beige grey to black background with or without beige red spots. Transverse bars on limbs are evident with fawn brown, but inconspicuous in other individuals. Venter in males shows olive yellow to terra brown tones, and in some females with platinum grey spots.

**Calls (Fig. 6).** The description of the call of *Pristimantis carchi* sp. nov. is published in Batallas, Márquez & Guayasamin (2025) (see *Pristimantis* sp. 2). In this work the principal acoustic information is provided in Table 1.

**Distribution and natural history (Figs. 1, 7).** *Pristimantis carchi* sp. nov. is a high-Andean species with a broad distribution across Carchi province, Ecuador. During the field of this study, specimens were collected from both the western and eastern flanks of the Andean range. The sites selected for collection were the Tufiño–Maldonado Road, Cascadas de Cartagena, Cerro La Bretaña, and Morán, at elevations ranging from 2,950 to 3,362 m. These sites correspond to the Northeastern Andean Montane Evergreen Forest, the Northwestern Andean Montane Evergreen Forest, and the Northeastern Andean High Mountain Evergreen Forest. These habitats are distinguished by high humidity and abundant vegetation, with trees reaching heights of 15 to 25 m, dense understory, and abundant epiphytes (especially orchids), ferns, and mosses (Ministerio del Ambiente del Ecuador (MAE), 2013).

Most individuals were observed during nighttime surveys, active among leaf litter, perched on fallen logs, within moss layers, adventitious roots, and on fern leaves or broad foliage. Some specimens were also recorded as active during daylight hours (Batallas, Márquez & Guayasamin, 2025). *Pristimantis carchi* sp. nov. is sympatric with *P. festae* and *P. cantrix* sp. nov. Although it occurs in areas with some degree of human intervention, its presence is restricted to sites where montane or high-Andean vegetative cover persists. It is rarely found in open landscapes used for livestock or agriculture, where *Pristimantis unistrigatus* is typically the dominant species (Batallas, Márquez & Guayasamin, 2025).

**Evolutionary relationships (Fig. 2).** In the *Pristimantis myersi* species group, it is closely related to a clade composed of *P. leoni* and a *Pristimantis* sp. (Franco-Mena et al., 2023).

**Etymology.** The specific epithet *carchi* refers to the province of Carchi, in northern Ecuador, where the type series was collected and where the species is abundant and widely distributed along both the eastern and western flanks of the northern Andes. More than a geographical reference, the name embodies the essence of Carchi: a high-altitude territory sheltered by cold and singular ecosystems. It evokes the abundance and resilience of a rich and diverse landscape, marked by ecological distinctiveness, deep territorial identity, and growing environmental pressures that threaten its conservation.

**Conservation status.** *Pristimantis carchi* sp. nov. is known from only four localities in the Carchi province, an area that has suffered deforestation and fragmentation because of agriculture and cattle farming. The current estimated extent of occurrence for the species is <5,000 km^2^. Therefore, following IUCN criteria to assess the current extinction risk of the species (Gärdenfors et al., 2001), we propose that *P. carchi* sp. nov. should be considered as Endangered (EN) B1 a, b (i, iii). We consider that future explorations could expand the known range and allow for a more accurate reassessment.

***Pristimantis levi* sp. nov**

*Pristimantis* sp. 18, Franco-Mena et al., 2023

*Pristimantis* sp. 5 Batallas, Márquez & Guayasamin, 2025

**LSID.** urn:lsid:zoobank.org:act:19332353-1131-421C-86B8-AD0E79E9986A

**Proposed standard English name:** Levi’s Rain Frog

**Proposed standard Spanish name:** Cutín de los Levi

**Figure 10:**
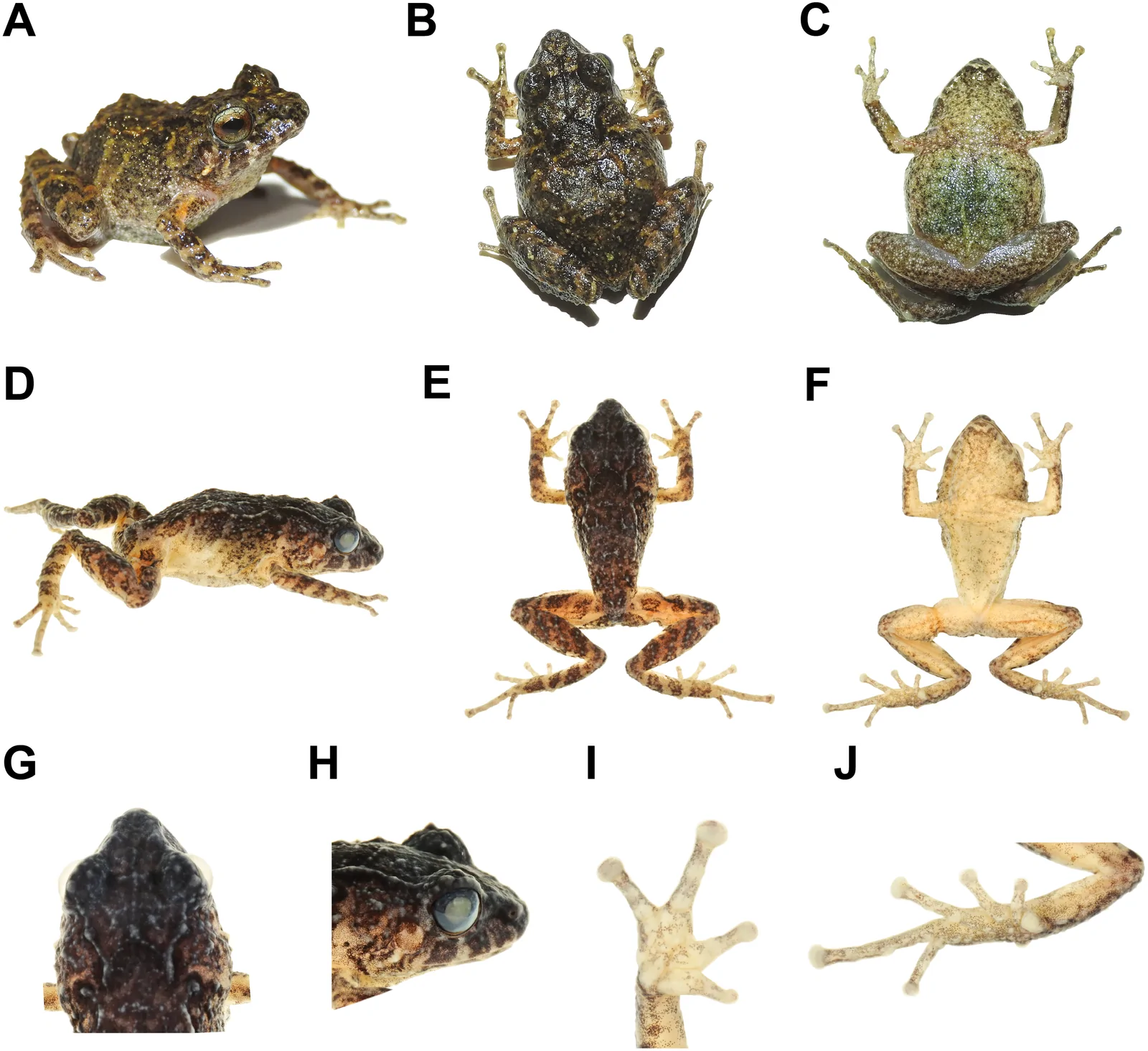
Holotype *Pristimantis levi* sp. nov. (ZSFQ 4498), adult male, SVL = 18.7 mm. (A–C) Live and (D–F) preserved photographs in lateral, dorsal and ventral view; (G) head in dorsal and (H) lateral view, (I) palmar surface, and (J) plantar surface. Photographs by Diego Batallas, Malki Bustos, and Daniela Franco-Mena.

**Holotype (Fig. 10).** ZSFQ 4498, adult male, collected on 25 March 2020 by Diego Batallas and Carlos Castro at Morán (0.768333 N, 78.051861 W; 2,895 m), Libertad, Espejo, Carchi province, Ecuador.

**Paratypes.** A total of eight specimens, including adult males (6) and one adult female, were collected in March 2020 at Morán, Libertad, Espejo, Carchi province, northern Ecuador. ZSFQ 4497, 4500, 4501 (adult males), with same data as the holotype. ZSFQ 4485 (adult male), collected on 19 March 2020 by Diego Batallas and Carlos Castro at Morán (0.768333 N, 78.051861 W; 2,784 m), Libertad, Espejo, Carchi province, Ecuador. ZSFQ 4489 (adult female), ZSFQ 4486, 4488 (adult males), collected on 19 March 2020 by Diego Batallas and Carlos Castro at Morán (0.768528 N, 78.055583 W; 2,829 m), Libertad, Espejo, Carchi province, Ecuador.

**Diagnosis.** *Pristimantis levi* sp. nov. can be distinguished from other *Pristimantis* by the following character combination: (1) skin on dorsum finely tuberculate with scattered larger tubercles; sinusoidal, ∨, or )( shaped scapular ridges, in some additional postocular ∨ shaped ridges follow from eyelid to scapula; venter areolate, discoidal fold at the level of the groin; (2) tympanic membrane differentiated from surrounding skin, tympanic annulus visible beneath the skin, rounded, posterodorsal slightly concealed beneath supratympanic fold, tympanum diameter varies from 50% to 80% of the eye diameter; (3) snout short, subacuminate in dorsal view, rounded in lateral view, without papilla at snout tip; (4) upper eyelid tuberculate, with two enlarged conical tubercles; upper eyelid wider than interorbital distance; cranial crests absent; (5) dentigerous process of vomer present, large, oval, positioned posterior to level of choanae, larger than choanae; (6) males lack vocal slits, subgular vocal sac, and nuptial pads; (7) Finger I shorter than Finger II; Finger I rounded without disc, Finger II rounded, or slightly elongated acuminate disc, Finger IV slightly elongated acuminate, or elliptical discs; (8) fingers with narrow laterals fringes; palmar tubercle bifid or divided; thenar tubercle oval, large as palmar tubercle; supernumerary tubercles not prominent, distributed on the basal parts of palm; hyperdistal subarticular tubercles not prominent, and basal subarticular tubercles rounded; (9) forearms with three small ulnar fold-like or conical tubercles; (10) heel bearing few low conical tubercles with or without an enlarged conical tubercle; outer edge of tarsus bearing 2–3 small subconical tubercles, inner tarsal fold present; (11) two metatarsal tubercles, inner oval or rounded, 1.36x–2.65x size of subconical outer tubercle; (12) toes with narrow lateral fringes; supernumerary tubercles round and small; Toe V shorter than Toe III, extending to the medial, or between medial and distal subarticular tubercles of Toe IV; (13) dorsal coloration khaki grey to grass green with black brown to bottle green irregular marks, limbs with khaki grey to grass green background, and chocolate brown transverse bars; ventral coloration agate grey background with olive green blotching; iris brown grey, stone grey or olive green, with a black brown, olive drab, or brown green median stripe; (14) SVL males 14.5–19.1 mm (mean 17.03 ± 1.89 mm, *n* = 7); female 19.9 mm (*n* = 1).

**Comparisons with other species (Fig. S3, Table S1).** According to Franco-Mena et al. (2023), *Pristimantis levi* is closely related to *Pristimantis gralarias* (Guayasamin, Arteaga-Navarro & Hutter, 2018), and *Pristimantis hectus* (Lynch & Burrowes, 1990); therefore, comparisons of morphological characteristics with these species are presented below. These three species share similar features such as ventral texture of skin (areolate), hyperdistal and basal subarticular tubercles (round), canthus rostralis (weakly concave), cranial crests (absent), lips (not flared), relative lengths of fingers (Finger I shorter than Finger II), lateral fringes of fingers and toes (present), and webbing (absent).

However, other characteristics show its singularities, the dorsal texture of skin is similar in these species; in *P. levi* is finely tuberculate with scattered larger tubercles; in *P. gralarias* is shagreen with numerous scattered low tubercles; and in *P. hectus* is granular, becoming tuberculate laterally. Eyelid tuberculate, in *P. levi* with two enlarged tubercles, *P. hectus* two to three subconical tubercles, and *P. gralarias* with no enlarged tubercles. Postrictal tubercles are large and conical in *P. levi* and *P. hectus*. Ulnar tubercles in *P. levi* are three conical or fold-like, in *P. gralarias* three low tubercles, and in *P. hectus* a row of three tubercles. Heel tubercles are present in these species, in *P. levi* has several low conical tubercles, *P. gralarias* has a small non-conical tubercle, and *P. hectus* a small tubercle. Dorsolateral folds in *P. levi* from posterior edge of scapula to sacrum, discontinuous and irregular; in *P. hectus* prominent ridges, in most individuals there is a pair of dorsolateral folds with little indication of other folds. The interocular fold in *P. levi* is mostly absent, in some individuals present as a row of low tubercles; in *P. hectus* head tubercles, with some tubercles fusing to form short ridges. The supratympanic fold in *P. levi* is present, and in *P. hectus* is thin. Paravertebral folds in *P. levi* and *P. hectus* are mostly absent, in some individuals there are ridges of low tubercles from posterior edge of scapula to anterior edge of sacrum. Occipital-scapular folds present in *P. levi* as sinusoidal, \/, or )( shaped scapular ridges, in some individuals additional postocular V shaped ridges follow from eyelid to scapula. Discoidal fold present in P. *levi* and *P. hectus*. Inner tarsal fold present in *P. levi* and *P. hectus*. Snout shape in *P. levi* and *P. hectus* are short, subacuminate in dorsal view, rounded in lateral view, without papilla at snout tip; in the other hand, in *P. gralarias* is short, rounded in dorsal and lateral profiles, with a small papilla at tip. Tympanum evident, in *P. levi* is round (1/2–4/5 of eye diameter), posterodorsal slightly concealed beneath supratympanic fold; in *P. hectus* (1/4–1/2 of eye diameter). Dentigerous processes of vomers present, in *P. levi* are large oval, positioned posterior to level of choanae; in *P. gralarias* are conspicuous, having triangular shape, well-separated from each other; and in *P. hectus* are oval. Vocal slits in *P. levi are not* visible, in *P. gralarias* unknown, and in *P. hectus* short posterolateral to the tongue. Vocal sac in males of *P. levi* and *P. hectus* subgular. Nuptial pads are absent in *P. levi* and *P. hectus*.

Discs in fingers of *P. levi* vary between fingers, as Finger I is rounded without disc, Finger II rounded, or slightly elongated acuminate disc, and Finger IV slightly elongated acuminate, or elliptical discs; in *P. gralarias* not expanded laterally (or only slightly expanded); and in *P. hectus* are present on Finger II–Finger IV, pads dilated, small, usually rounded apically, Finger I without disc and pad scarcely dilated, pointed, Finger II with smaller pad, dilated pointed in many, rounded in others. In *P. levi* fingers with narrow laterals fringes; palmar tubercle bifid or divided; thenar tubercle oval, large as palmar tubercle; supernumerary tubercles, distributed on the basal parts of palm; hyperdistal subarticular tubercles not prominent, and basal subarticular tubercles rounded and *P. lasgralarias* round, simple, with moderate-sized. Toe V shorter than Toe III, extending to the medial, or between medial and distal subarticular tubercles of Toe IV in *P. levi*; and Toe V about the same length as Toe III in *P. gralarias*. Webbing absent in *P. levi* and *P. gralarias*. Discs of toes in *P. levi* are rounded, narrow, and slightly elongated acuminate; in *P. gralarias* are not expanded laterally, pads with clearly defined circumferential groove; and in *P. hectus* are expanded and lanceolate. Tarsal tubercles in *P. levi* with two or three conical outer tubercles, in *P. gralarias* with four small non conical tubercles, and in *P. hectus* with a row of larger tubercles than heel along the outer edge of tarsus. In terms of plantar tubercles *P. levi* shows two metatarsal tubercles, inner oval or rounded (smaller than *P. gralarias* and *P. hectus*) 1.3–2.5 times subconical outer tubercle; in *P. gralarias*, inner metatarsal tubercle conspicuous, oval, four to five times round outer metatarsal tubercle; and in *P. levi* is bifid or divided, if divided in two segments oval or round tubercles, if divided in three to four segments rounded tubercles, thenar tubercle oval almost as large as palmar tubercle; in *P. gralarias* bifid distally and well differentiated; and in *P. hectus* bifid or divided in two segments, larger than oval thenar tubercle four times subconical outer metatarsal tubercle. Supernumerary tubercles are small in *P. levi*, *P. gralarias*, and *P. hectus*; but in *P. levi*, and *P. gralarias* also bulky tubercles.

**Description of the holotype (Fig. 10).** Adult male (ZSFQ 4498), head longer than wide, length 37.9% SVL, width 37.4% SVL; snout long and subacuminate in dorsal and lateral view. Eye nostril distance 10.16% SVL; canthus rostralis concave, nostrils directed laterally; interorbital area flat, thin than the upper eyelid, upper eyelid represents 62.9% interorbital distance; cranial crests absent; presence of a W-shaped scapular fold in the occipital/scapular region, presence of two pairs of prominent subconic tubercles on the occipital and scapular regions; upper eyelid with one prominent subconical tubercle on the center of eyelid, surrounded by several lower subconical tubercles (all tubercles reduced by preservation effects); tympanic membrane differentiated from surrounding skin, tympanic annulus visible beneath the skin, rounded, laterally oriented, tympanum diameter 51.8% of the eye diameter, postrictal tubercles present; moderate size in choanae, with a round-shaped outline, not concealed by palatal shelf of maxilla; dentigerous process of vomer present, large oval, moderately separated, posteromedial to choanae; oval tongue longer. Skin on dorsum finely tuberculate to tuberculate, with “W” shaped scapular folds and dermal ridges, with rounded tubercles widespread; dorsolateral folds absent; skin on venter coarsely areolate; discoidal fold at the level of the groin; anal ornamentation present, with several rounded tubercles. Forearms slender with subconical ulnar tubercles and a row of subconical tubercles along the anterior edge (reduced or less evident in preservation effects); Fingers with fine lateral fringes, palmar tubercle heart-shaped, bilobed, approximately one and a half the size of thenar tubercle; hyperdistal subarticular tubercles not prominent and basal subarticular tubercles rounded, with supernumerary tubercles at the base of each digit; digital pads truncated and expanded, twice as wide of the digit in Finger III, Finger IV, on fingers I and II is slightly wide than digit; all fingers have digital pads defined by circumferential grooves. Hindlimbs slender, tibia length 50.78% SVL, two subconical tubercles on the heel, a row of subconical tubercles on the outer edge of tarsus; inner tarsal fold present; Toes with fine lateral fringes, without digital webbing, digital pads of the toes expanded, Toe IV and Toe V twice width than digits, Toe I, Toe II, Toe III slightly expanded than digit; low and rounded subarticular tubercles, supernumerary tubercles weakly defined rounded; metatarsal tubercles present, inner oval three times than outer tubercle that is rounded; Toe V longer than Toe III, reaching to the base of distal subarticular tubercle of Toe IV.

**Measurements (in mm) of the holotype (ZSFQ 4498).** SVL = 18.7, HL = 7.1, HW = 7.0, IOD = 1.9, UEW = 2.0, EL = 2.7, END = 1.9, TY = 1.4, IND = 2.1, UAL = 4.0, FLL = 4.1, HAL = 5.3, Finger I = 2.4, Finger II = 3.0, Finger III DW = 0.9, THL = 9.1, TL = 9.4, FL = 9.2, and Toe IV DW = 1.0.

**Color of holotype in life (Fig. 10).** Dorsum dark brown, background with irregular light brown to yellowish cream transverse markings, with scattered cream spots; dorsal surface of forelimbs and hindlimbs with brownish-black transverse bars; flanks cream with yellowish cream diagonal stripes; head with subocular and labial brown diagonal bars. Belly yellowish cream with coppery tones on forearms, marbled with dark brown; throat yellowish cream with a brown “W” mark at end of snout; groin and hidden surfaces of thigh yellowish cream. Iris coppery/yellow with fine black reticulations.

**Color of holotype in ethanol (Fig. 10).** Dorsum brown with irregular yellowish cream markings; dorsal surfaces of hind and forelimbs yellowish cream with brown crossbars; with subocular and labial brown diagonal bars; flanks yellowish cream with dark brown diagonal stripes; groin and concealed surfaces of hind limbs cream. Belly and throat yellowish cream marbled with dark brown, throat with light brown “W” mark; ventral surfaces of calf marbled with brown spots.

**Figure 11:**
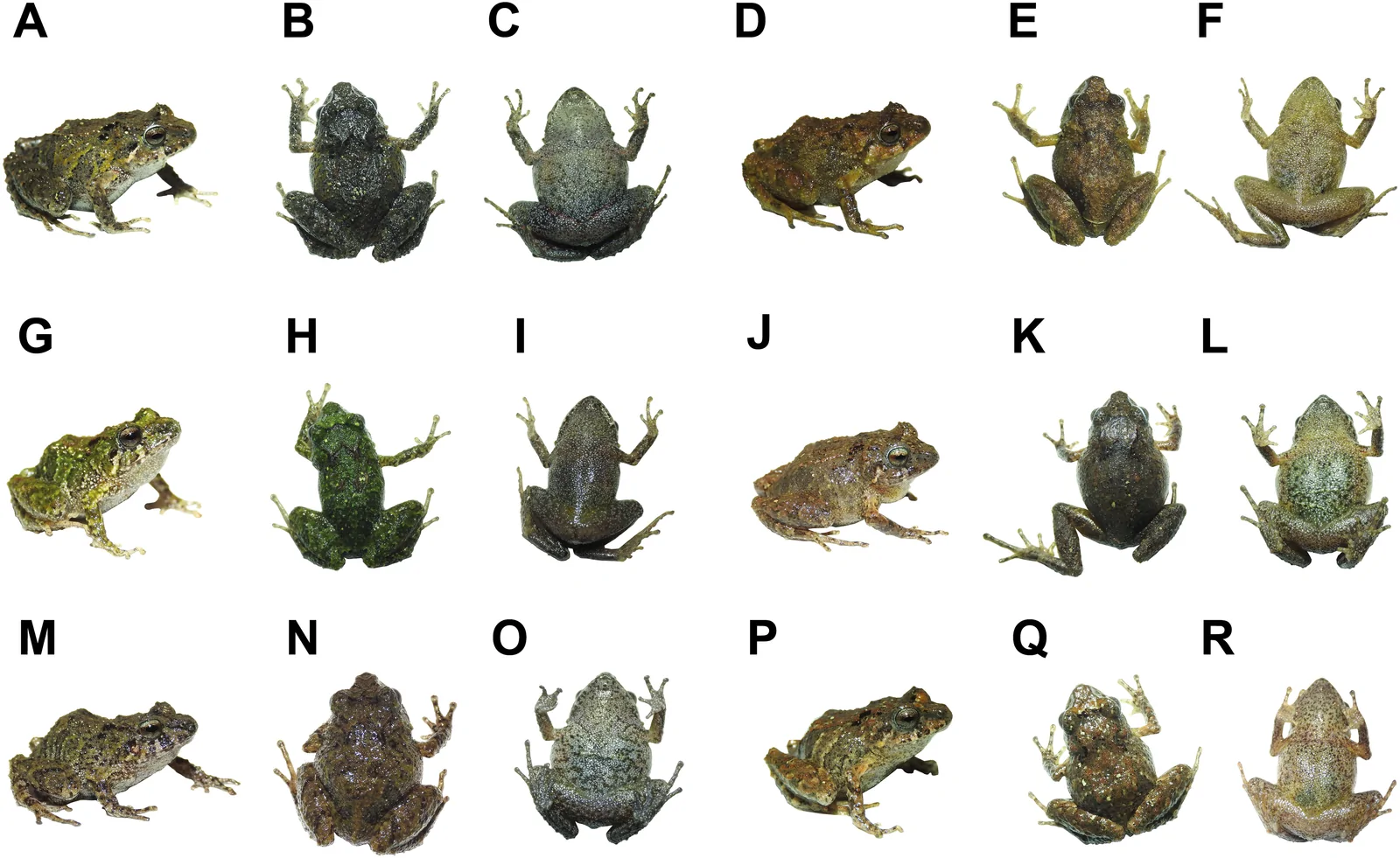
Color variation in life of *Pristimantis levi* sp. nov. Photographs in lateral, dorsal, and ventral view. (A–C) ZSFQ 4489 female, (D–F) ZSFQ 4485 male, (G–I) ZSFQ 4486 male, (J–L) ZSFQ 4497 male, (M–O) ZSFQ 4500 male, (P–R) ZSFQ 4501 male. Photographs by Diego Batallas.

**Variation.** Morphometric variation is shown in Appendix S1 and Fig. 4. *Pristimantis levi* sp. nov. shows variation in color (Figs. 11, S6). Interorbital bar, if present, may show coloration from graphite black to black green. Canthal stripe and postorbital stripe display different tones of brown, such as olive drab, black brown, sepia brown, or bottle green. Labial bars in olive drab, brown green, or slate grey. Dorsal coloration varies from patterns of khaki grey, grey, olive green or grass green background; with irregular marks of black brown, or bottle green. Axilla and groin exhibit green tones such as olive yellow, reed green, or green brown. Transverse bars on limbs are evident with chocolate brown, khaki grey, or bottle green. Venter patterns show agate grey, green beige, or pebble grey background tones, with olive green, green grey, chrome green or black olive blotching.

**Calls (Fig. 6).** The description of the call of *Pristimantis levi* sp. nov. is published in Batallas, Márquez & Guayasamin (2025) (see *Pristimantis* sp. 5). In this work the principal acoustic information is provided in Table 1.

**Distribution and natural history (Figs. 1, 7).** *Pristimantis levi* sp. nov. is a high-Andean species with a restricted distribution in Morán, occurring at elevations between 2,784 and 2,895 m on the western slopes of the Andes in northern Ecuador. It inhabits Northwestern Andean Montane Evergreen Forest (Ministerio del Ambiente del Ecuador (MAE), 2013), a habitat characterized by dense vegetation cover, medium to tall trees, abundant epiphytes, and a dense understory of herbs and shrubs. This species is crepuscular and nocturnal. Individuals were observed perched in grasslands, leaf litter, and shrubby vegetation between 40 and 80 cm above the ground. *Pristimantis levi* sp. nov. typically inhabits open high-Andean environments, including grasslands, carrizo thickets, and transitional zones under livestock use. In contrast to other members of the *Pristimantis myersi* group, which occupy forested areas with dense and tangled understory, *P. levi* sp. nov. shows no evidence of sympatry with them. Its presence is restricted to degraded sites, such as roadside edges, and it is rarely found in forested habitats (Batallas, Márquez & Guayasamin, 2025).

**Evolutionary relationships (Fig. 2).** In the *Pristimantis myersi* species group, it is closely related to two clades composed of *P. gralarias*, *P. hectus, P. onorei*, and *P. lucidosignatus* (Franco-Mena et al., 2023).

**Etymology.** The specific epithet *levi* is a recognition of Jaime Levy and his family, whose work has significantly contributed to conservation in northern Ecuador and southern Colombia through a deeply community-centered vision. Their efforts have transcended borders, generating territorial planning processes that have reclaimed collective rights, with tangible impact across economic, social, and environmental spheres. This species pays tribute to those who have believed with conviction and wisdom that it is the youth and the people who inhabit their territories who will protect and defend their biodiversity, not through imposition, ego, or arrogance, but through humility, education, awareness, and leadership.

**Conservation status.** *Pristimantis levi* sp. nov. is known from only one locality in the Carchi province, an area that has suffered deforestation and fragmentation because of agriculture and cattle farming. The current estimated extent of occurrence for the species is <100 km^2^. Therefore, following IUCN criteria to assess the current extinction risk of the species (Gärdenfors et al., 2001), we propose that *P. levi* sp. nov. should be considered as Critically Endangered (CR) B1 a, b (i, iii).

***Pristimantis marquezi* sp. nov**

*Pristimantis* sp. 15, Franco-Mena et al., 2023

*Pristimantis* sp. 4 Batallas, Márquez & Guayasamin, 2025

**LSID.** urn:lsid:zoobank.org:act:4472300F-D4EA-4A64-8571-249E3C3C1077

**Proposed standard English name:** Márquez Rain Frog

**Proposed standard Spanish name:** Cutín de Márquez

**Figure 12:**
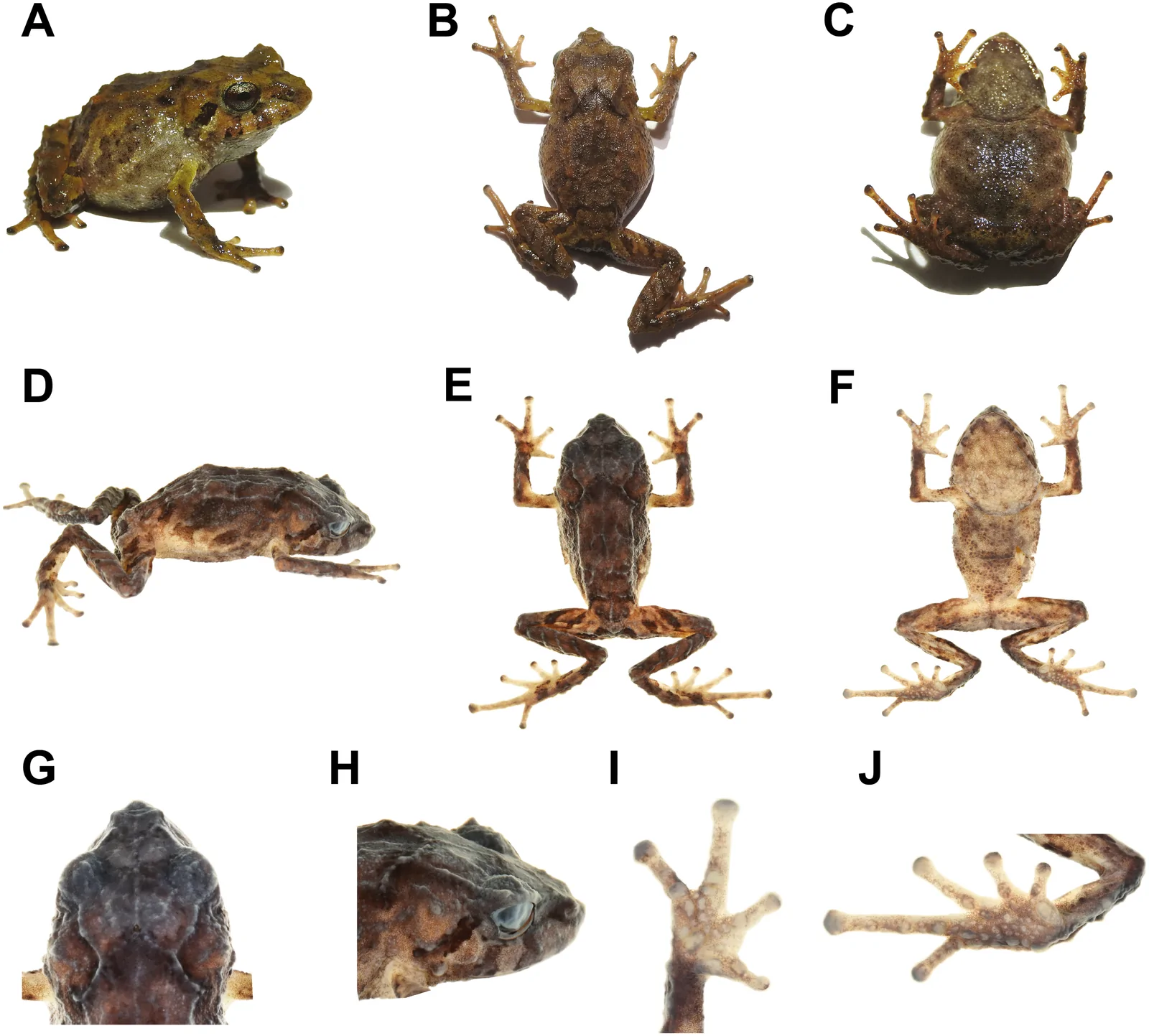
Holotype *Pristimantis marquezi* sp. nov. (ZSFQ 4429), adult male, SVL = 16.1 mm. (A–C) Live and (D–F) preserved photographs in lateral, dorsal and ventral view; (G) head in dorsal and (H) lateral view, (I) palmar surface, and (J) plantar surface. Photographs by Diego Batallas, Malki Bustos, and Daniela Franco-Mena.

**Holotype (Fig. 12).** ZSFQ 4429 (adult male), collected on 15 December 2020 by Diego Batallas, Franklin Rodríguez, and José Rodríguez at San Francisco de Pioter (0.679611 N, 77.797917 W; 3,411 m), Pioter, Tulcán, Carchi province, northern Ecuador.

**Paratypes.** A total of 12 specimens, including adult males (9) and females (2), were collected between 14 December 2020 and 10 June 2021 in a single locality in northern Ecuador. ZSFQ 4428, 4430 (adult males), collected on 15 December 2020, with same data as the holotype; ZSFQ 4426, 4427 (adult males), collected on 14 December 2020; ZSFQ 4431, 4432, 4433 (adult males), collected on 16 December 2020; ZSFQ 4434, 4435 (adult males), ZSFQ 4436 (adult female), collected on 17 December 2020; ZSFQ 4463 (adult female), collected on 10 June 2021, all from San Francisco de Pioter, Pioter, Tulcán, Carchi province, northern Ecuador.

**Diagnosis.** *Pristimantis marquezi* can be distinguished from other *Pristimantis* by the following character combination: (1) skin on dorsum smooth tuberculate, with scattered larger tubercles; low sinusoidal, W, or )( shaped scapular ridges, in some postocular ridges follows from eyelid tubercles to scapula, and then to sacrum, as part of weakly developed dorsolateral folds; venter areolate to coarsely areolate, discoidal fold at the level of the groin; (2) tympanic membrane differentiated from surrounding skin, tympanic annulus visible beneath the skin, rounded, posterodorsal slightly concealed beneath supratympanic fold, tympanum diameter varies from 60% to 80% of the eye diameter; (3) snout short, subacuminate or acutely rounded in dorsal view, rounded in lateral view, with a small rostral papillae at snout tip; (4) upper eyelid tuberculate, with two enlarged conical tubercles; upper eyelid wider than interorbital distance; cranial crests absent; (5) dentigerous process of vomer low, obliquely angled posteromedial, barely visible until absent; (6) males lack vocal slits, subgular vocal sac, and nuptial pads; (7) Finger I shorter than Finger II; Finger I and FinII discs narrow and rounded, Finger III and Finger IV discs slightly expanded and elliptical; (8) fingers with narrow laterals fringes; palmar tubercle bifid; thenar tubercle barely visible and oval, larger than palmar tubercle; supernumerary tubercles, distributed on the basal parts of palm; hyperdistal subarticular tubercles not prominent, and basal subarticular tubercles prominent and rounded; (9) forearms with three small ulnar conical or fold-like tubercles; (10) heel bearing few low conical tubercles with an enlarged conical tubercle; outer edge of tarsus bearing three triangle or subconical tubercles, inner tarsal fold present; (11) two metatarsal tubercles, inner oval, 1.5x–1.8x size of rounded outer tubercle; (12) toes with narrow lateral fringes; supernumerary tubercles round, and small; Toe V shorter than Toe III, extending to medial subarticular tubercles of Toe IV; (13) dorsal coloration terra brown to black background with brown beige irregular marks, limbs with terra brown to black background, and chocolate brown or black red transverse bars; ventral coloration black brown to chocolate brown background with beige grey or brown green blotching; iris pastel green to brown grey, with an olive drab, or black median stripe; (14) SVL males 11.3–16.6 mm (mean 14.51 ± 1.62 mm, *n* = 9); females 21.0– 21.5 mm (mean 21.22 ± 0.37 mm, *n* = 2).

**Comparisons with other species (Fig. S3, Table S1)**. According to Franco-Mena et al. (2023), *Pristimantis marquezi* (sp. 15) is closely related to *Pristimantis munozi* (Rojas-Runjaic, Delgado & Guayasamin, 2014), *Pristimantis ocreatus* (Lynch, 1981), and *Pristimantis cantrix* (sp. 14); therefore, comparisons of morphological characteristics with these species are presented below. These four species share similar features such as dorsal texture of skin (finely areolate, tuberculated, or rough to slightly smooth), eyelid tuberculated, hyperdistal and basal subarticular tubercles (round and bulky), discoidal fold (present), cranial crests (absent), relative lengths of fingers (Finger I shorter than Finger II), and webbing (absent).

However, other characteristics show its singularities, ventral skin is similar in *P. arquezi, P. cantrix*, and *P. ocreatus* with areolate to coarsely areolate texture; *P. munozi* also shares this feature but may exhibit a smooth to slightly areolate texture on the throat. Postrictal tubercles in *P. cantrix* are small to moderately sized, and conical; while in *P. marquezi* are long and conical; *P. ocreatus* has a non-conical postrictal tubercle. Ulnar tubercles in *P. marquezi* tree conical or fold-like tubercles, *P. cantrix* exhibit tree conical tubercles, and *P. ocreatus* have no distinctive tubercles. Dorsolateral folds in *P. marquezi, P. munozi, P. ocreatus*, and *P. cantrix* are present and may be from posterior edge of scapula to sacrum, but in *P. cantrix* could be from posterior edge of eyelid to sacrum also. Lateral folds are occasionally present in some individuals of *P. marquezi* as rows of low conical tubercles, and in *P. cantrix* as rows of low ridges and tubercules. Interocular fold absent in P. *munozi, P. ocreatus*, and *P. marquezi*; but *P. cantrix* could have a row of low tubercles. Supratympanic fold present in *P. marquezi* and *P. cantrix*; not prominent in *P. ocreatus*. Paravertebral folds are mostly absent but sometimes present as low irregular ridges in *P. marquezi* and *P. ocreatus*, and low ridges in *P. cantrix*. Middorsal fold absent in *P. marquezi*, sometimes present as a row of low tubercles from frontoparietal to sacrum in *P. cantrix*. Occipital-scapular folds present in *P. marquezi* and *P. cantrix* as sinusoidal, W, or )( shaped scapular ridges, and in some individuals’ postocular ridges as part of dorsolateral folds; *P. munozi* also has dorsolateral folds. Thoracic fold present in *P. marquezi* and *P. cantrix*. Inner tarsal fold present in *P. marquezi, P. munozi, P. ocreatus*, and *P. cantrix*. Snout shape in *P. marquezi* is subacuminate or acutely rounded in dorsal view, rounded in lateral view, with small rostral papilla at snout tipin; *P. cantrix* is acutely rounded in dorsal view, rounded in lateral view, with a small rostral papilla at snout tip; in *P. munozi* is short, acuminate in dorsal view, rounded in profile; in *P. ocreatus* is very short, round in dorsal and lateral profile. Canthus rostralis in *P. marquezi, P. munozi*, and *P. cantrix* is weakly concave; in *P. ocreatus* is rounded to obtuse. Lips not flared in *P. marquezi, P. ocreatus*, and *P. cantrix*. Tympanum in *P. marquezi* is evident, round (3/5–4/5 of eye diameter), posterodorsal slightly concealed beneath supratympanic fold; in *P. cantrix* is evident, round (3/5–9/10 of eye diameter), posterodorsally slightly concealed beneath supratympanic fold; in *P*. *munozi* is small, but well-defined (1/2–2/5 of eye diameter), slightly higher than long, posterodorsally concealed beneath skin; and in *P. ocreatus* is concealed beneath skin. Vocal slits in *P. marquezi* are not visible, whereas in *P. cantrix* are present, in *P. munozi* prominent, and in *P. ocreatus* short. Vocal sac in *P. marquezi* and *P. cantrix* subgular, and in *P. ocreatus* internal. Vocal sac in *P. marquezi* and *P. cantrix* subgular, and in *P. ocreatus* internal. Nuptial pads in *P. marquezi, P. munozi*, and *P. cantrix* absent.

Lateral fringes of fingers in *P. marquezi* and *P. cantrix* are narrow, in *P. munozi* preaxial keel on Finger II–Finger III, and absent in *P. ocreatus*. Discs of fingers in *P. marquezi* Finger I and Finger II discs are narrow and rounded, while Finger III and Finger IV are slightly expanded and elliptical; in *P. cantrix* are narrow, rounded, slightly elongated acuminate or expanded; in *P. munozi* are narrow and rounded; and in *P. ocreatus* as long as wide on Finger III and Finger IV. Toe V shorter than Toe III, extending to the medial subarticular tubercles of Toe IV in *P. marquezi* and *P. cantrix*; whereas *P. munozi* has Toe V slightly longer than Toe III, and not extending to the proximal edge of the distal subarticular tubercle of Toe IV. Lateral fringes of toes in *P. marquezi* and *P. cantrix* are narrow; whereas in *P. munozi* and *P. ocreatus* are absent. Discs on toes in *P. marquezi* and *P. cantrix* are rounded, narrow, slightly elongated acuminate, while in *P. munozi* are slightly expanded. In *P. marquezi* hyperdistal subarticular tubercles not prominent, and basal subarticular tubercles prominent and rounded and in *P. munozi* Round, small to large, bulky. Heels in *P. marquezi*, *P*. *munozi*, and *P. cantrix* are described as tuberculated with few, low tubercles; *P. ocreatus* doesn’t have enlarged or distinctive heel tubercles. Tarsal tubercles in *P. marquezi*, *P. cantrix* and *P. munozi* are present, but with its own singularities, such as, *P. marquezi* three small triangular or fold-like tubercles, *P. munozi* have four to five-fold-like tubercles, *P. cantrix* three small conical tubercles; in contrast, *P. ocreatus* have no enlarged or distinctive tarsal tubercles. In terms of plantar tubercles *P. marquezi*, *P. munozi*, P. *cantrix*, and *P. ocreatus* shows two metatarsal tubercles, inner oval and larger than outer. *P. cantrix* shows the shorter difference between metatarsal tubercles size, with inner tubercle 1.3–1.5 times outer tubercle, followed by *P. marquezi* with 1.5–1.8 times bigger, *P. munozi* about two times bigger, and *P. ocreatus* 2–3 times size of outer tubercle. Palmar tubercle is bifid in *P. marquezi, P. cantrix*, and *P. ocreatus*, but *P. marquezi* only oval tubercle, or divided ovals, whereas *P. cantrix* may present single round or oval tubercle. Thenar tubercle is almost as large as palmar tubercle. In *P. marquezi* thenar tubercle may be barely visible, and in *P. cantrix* palmar tubercles may be barely visible. Supernumerary tubercles in *P. marquezi* and *P. cantrix* are round, small, and bulky, whereas in *P. ocreatus* are indistinct.

**Description of the holotype (Fig. 12).** Adult male (ZSFQ 4429), head moderately wide than longer, length 37.26% SVL, width 37.8% SVL; snout rounded with small rostral papillae at tip of the snout, snout rounded in dorsal and lateral view. Eye nostril distance 9.9% SVL; canthus rostralis and loreal region slightly concave; nostril slightly protuberant oriented laterally; interorbital area flat, with tubercles, wider than the upper eyelid, upper eyelid represents 80.0% interorbital distance; cranial crests absent; occipital fold defined by the presence of two rows of prominent dermal folds in form of sinusoidal ridges; row of small, rounded tubercles from the tip of the snout to the interorbital region; upper eyelid with one subconical tubercles (rounded in preservative), surrounded by several lower subconical tubercles (reduced by preservation effects); rounded tubercles scattered on the canthus rostralis and loreal region; tympanic membrane differentiated from surrounding skin, tympanic annulus visible beneath the skin, rounded, laterally oriented, tympanum diameter 63.1% of the eye diameter, postrictal tubercles present, low; small choanae, rounded in outline, not covered by the palatal floor of maxilla; dentigerous process of vomer present oblique in outline; oval tongue. Skin on dorsum finely tuberculate to tuberculate, with “W” shaped scapular folds and dermal ridges, with rounded tubercles widespread; venter areolate with some pustules and scattered warts, discoidal fold at the level of the groin; anal ornamentation present, with several rounded tubercles. Forearms slender with a row of subconical tubercles along the anterior edge (in preservation effects); Fingers with fine lateral fringes, palmar tubercle heart-shaped, bilobed, approximately one and a half the size of thenar tubercle; hyperdistal subarticular tubercles rounded and not prominent, basal articular tubercle prominent with supernumerary tubercles at the base of each digit; digital pads truncated and expanded, twice as wide of the digit in fingers III, IV, on fingers I and II is slightly wide than digit; all fingers have digital pads defined by circumferential grooves. Hindlimbs slender, tibia length 42.2% SVL, one subconical tubercle on the heel, a row of subconical tubercles on the outer edge of tarsus; inner tarsal fold present; Toes with fine lateral fringes, without digital webbing, digital pads of the toes expanded, Toes IV and III twice width than digits, Toes I, II, V slightly expanded than digit; low and rounded subarticular tubercles, supernumerary tubercles weakly defined rounded; metatarsal tubercles present, inner oval three times than outer tubercle that is rounded; Toe III longer than V, reaching to the base of distal subarticular tubercle of Toe IV.

**Measurements (in mm) of the holotype (ZSFQ 4429).** SVL = 16.1, HL = 6.0, HW = 6.1, IOD = 2.0, UEW = 1.6, EL = 1.9, END = 1.6, TY = 1.2, IND = 1.9, UAL = 3.2, FLL = 4.0, HAL = 4.2, Finger I = 2.1, Finger II = 2.1, Finger III DW = 0.7, THL = 6.8, TL = 6.8, FL = 7.3, and Toe IV DW = 0.8.

**Color of holotype in life (Fig. 12).** Dorsal surfaces light brown to brown, dorsolateral lines brown, laterally lighter with dark brown shades, and forelimbs yellowish green; delineated by brown reticulations. The dorsal surfaces of the legs are brown with contrasting dark brown bars. Its belly is areolate brown with dark brown spicules, the gular region has cream brown tints. The iris is greenish-yellowish copper with a mid-horizontal bar of dark red copper color and a copper ring around the pupil with black reticulations.

**Color of holotype in ethanol (Fig. 12).** Dorsal surfaces are brown, dorsolateral lines light brown, laterally lighter with dark brown shades and on the forelimbs a yellowish cream color. The dorsal surfaces of the legs are brown with dark contrasting bars. Its belly is yellowish cream areolate with dark brown spots, the gular region has cream brown tints; ventral surfaces of the forelimb marbled with brown spots.

**Calls (Fig. 6).** The description of the call of *Pristimantis marquezi* sp. nov. is published in Batallas, Márquez & Guayasamin (2025) (see *Pristimantis* sp. 4). In this work the principal acoustic information is provided in Table 1.

**Figure 13:**
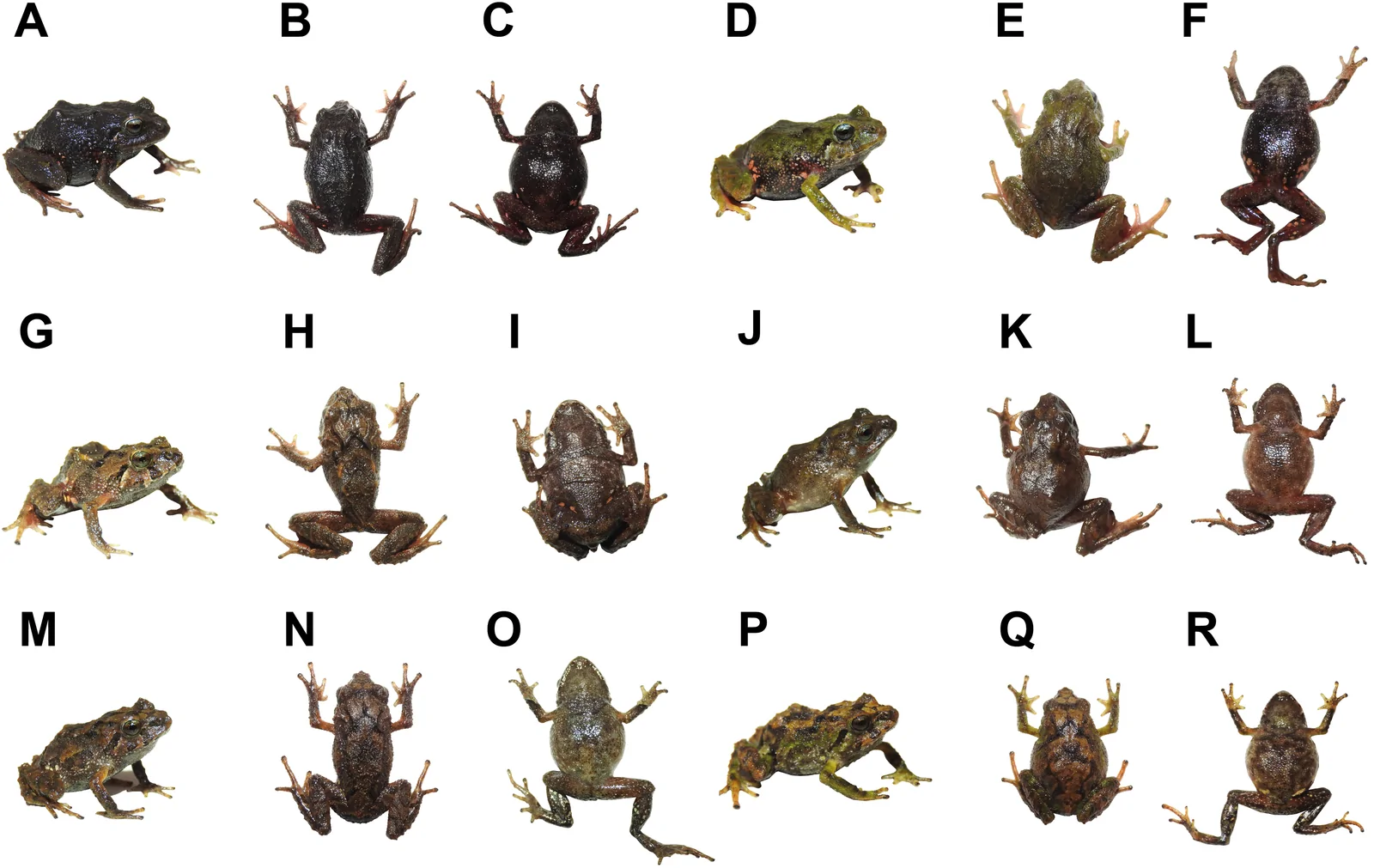
Color variation in life of *Pristimantis marquezi* sp. nov. Photographs in lateral, dorsal, and ventral view. (A–C) ZSFQ 4436 female, (D–F) ZSFQ 4463 female, (G–I) ZSFQ 4435 male, (J–L) ZSFQ 4428 male, (M–O) ZSFQ 4430 male, (P–R) ZSFQ 4432 male. Photographs by Diego Batallas.

**Variation.** Morphometric and acoustic variation is shown in Appendix S1 and Fig. 4. *Pristimantis marquezi* sp. nov. shows variation in color (Figs. 13, S7). Interorbital bar terra brown to olive drab. The canthal stripe and postorbital stripe display tones of black to grey brown. Labial bars, if present in brown grey, or brown. Dorsal coloration varies from patterns of terra brown, brown grey, green brown, or black background; with irregular marks of black, or brown beige. Axilla and groin exhibit grey and brown tones such as khaki grey, black brown, or chocolate brown background, in males with ivory blotching, in females with beige red or salmon pink blotching and spots. Transverse bars on limbs are evident with chocolate brown or black red. Venter patterns show black brown, khaki grey, or chocolate brown background tones, with beige grey, grey brown, or brown green blotching.

**Distribution and natural history (Fig. 1).** *Pristimantis marquezi* sp. nov. is a high-Andean species with a restricted distribution in San Francisco de Pioter, occurring at 3,411 m on the western slopes of the Andes in northern Ecuador. It inhabits Northwestern Andean Montane Evergreen Forest (Ministerio del Ambiente del Ecuador (MAE), 2013), a high-elevation ecosystem characterized by medium to tall trees (up to 20 m), abundant epiphytes and bryophytes, and a dense understory of herbaceous vegetation. Unlike gradual ecotonal transitions, this forest lies in a zone where montane vegetation and páramo coverage meet abruptly, resulting in a heterogeneous landscape of microhabitats shaped by elevation, humidity, and vegetation structure. This species is crepuscular and nocturnal. Individuals have been observed perched in leaf litter, dense undergrowth, hollow trunks, and at the base of adventitious tree roots, as well as on low vegetation between 20 and 60 cm above the ground. *P. marquezi* sp. nov. inhabits forest ecosystems with dense understory. It is very rare in páramo environments, where only *Pristimantis festae* has been recorded (Batallas, Márquez & Guayasamin, 2025).

**Evolutionary relationships (Fig. 2).** In the *Pristimantis myersi* species group, it is closely related to *P*. *cantrix* sp. nov., *P. munozi*, and *P. ocreatus* (Franco-Mena et al., 2023).

**Etymology.** The specific epithet marquezi honors Rafael Ignacio Márquez Martínez de Orense, a Spanish biologist whose work with animal sounds has made lasting contributions to zoology. As founder of the Fonoteca Zoológica de Madrid, he has built one of the most comprehensive archives of animal vocalizations, enabling major advances in ecology, taxonomy, and behavior, and offering a sonic window into the natural world. His scientific legacy, wisdom, and distinctive sense of humor have inspired new generations of zoologists to forge deeper connections with the organisms they study. His work reminds us to listen because nature, and all its beings, have a voice.

**Conservation status.** *Pristimantis marquezi* sp. nov. is known from only one locality in the Carchi province, it is found only in forest areas where the undergrowth is dense and well preserved. It has not been recorded in pastures or open areas, indicating a high sensitivity to habitat transformation. This location represents one of the last remaining forests in Carchi that connect the eastern and western Andes and faces strong pressure from agricultural and livestock activities. The current estimated extent of occurrence for the species is <100 km^2^. Therefore, following IUCN criteria to assess the current extinction risk of the species (Gärdenfors et al., 2001), we propose that *P. marquezi* sp. nov. should be considered as Critically Endangered (CR) B1 a, b (i, iii).

**Changes in vegetation cover.** The spatio-temporal analysis of vegetation in Carchi province indicates that in 1985, the area covered by vegetation was 195,349 hectares. The highest density of vegetation was observed in the western foothills, extending from the high montane forests to the lowlands of the Ecuadorian Chocó. Also, in the eastern foothills, important remnants of high montane forest vegetation persisted. In 2022, the vegetation area decreased to 186,123 hectares. Specifically, there is a gross loss of 25,501 hectares of vegetation by 2022 compared to 1985, with the eastern foothills being the most affected, followed by the lowlands of the Ecuadorian Chocó (Fig. 1). This reduction significantly affects the habitat of the new species: *P. carchi* sp. nov., *P. cantrix* sp. nov., and *P. marquezi* sp. nov., whose distribution is concentrated in these areas. While for the habitat of *P. levi* sp. nov., the loss of vegetation has slowed down. On the other hand, the analysis shows that in 2022 there was a gain of 16,275 hectares of vegetation in the province, characterized by small and dispersed patches. This suggests that there has been a net loss of 9,226 hectares of vegetation in the province of Carchi between 1985 and 2022.

## Discussion

This article presents the description of new frog species that belong to the *Pristimantis myersi* group. These species were obtained during systematic explorations of the high Andean ecosystems of Carchi Province, northern Ecuador. These sampling campaigns have resulted in one of the most complete collections of specimens from this group in the region and have contributed to a methodical and detailed record of these ecosystems. It is important to note, however, that records of the *Pristimantis myersi* group in Carchi are neither recent nor isolated. They are the result of decades of sustained research, reflected in multiple publications and projects (*e.g*., Lynch & Duellman, 1997; Frolich et al., 2005; Boada & Campaña, 2008; Yánez-Muñoz et al., 2020; Franco-Mena et al., 2023; Yánez-Muñoz et al., 2025).

This accumulation of knowledge has enabled the construction of a solid foundation for understanding the herpetological diversity of a historically understudied group. The taxonomic delimitation of the *Pristimantis myersi* group has proven challenging due to its cryptic nature and the morphological convergence among closely related species (Guayasamin et al., 2015; Franco-Mena et al., 2023; Yánez-Muñoz et al., 2025), which has hindered their recognition both in the field and within collections. This challenge has led to a prolonged accumulation of undetermined specimens and a systematic underestimation of their diversity. The phylogenetic revision by Franco-Mena et al. (2023) redefined the boundaries of the *P*. *myersi* group *sensu* stricto and formalized the subgenus *Trachyphrynus*, recognizing multiple candidate species, including the four described herein. These species, collected during systematic surveys in Carchi, were essential to that process, and their inclusion in the phylogenetic matrix allowed for the establishment of robust evolutionary relationships among previously unrecognized lineages. Their formalization is not only supported by phylogenetic structure, but also by geographic, behavioral, and morphological evidence, providing a comprehensive curatorial framework for each taxon.

These species remained undocumented for decades due to inadequate diagnoses and the lack of sufficient comparative material, including specimens and genetic sequences. The habitats where they occur are not included in the National System of Protected Areas (Boada & Campaña, 2008; Yánez-Muñoz et al., 2020). Although local protection initiatives led by sectional governments, communities, and territorial actors are in place, they lack legal recognition and operational capacity. The loss of 9,226 hectares of vegetation between 1985 and 2022 has intensified the isolation of mountain relicts, undermined ecological connectivity and potentially promoting cryptic speciation in species with extremely restricted ranges (Hutter & Guayasamin, 2015; Arteaga et al., 2016; Páez & Ron, 2019). In addition, habitat loss significantly increases the risk of species extinction. Newly described species are not evenly distributed throughout the forest but depend on well-preserved understory vegetation. However, these habitats are increasingly fragmented by seemingly minor activities, such as the opening of roads, trails, or access routes, both by local communities and tourism. Our results indicate that the highest loss of vegetation in the province of Carchi is concentrated in the eastern foothills, especially in San Francisco de Pioter, Cascadas de Cartagena, Virgen Negra, Bosque de Los Arrayanes, Cerro La Bretaña, and Loma La Esperanza. This reduction in vegetation cover is mainly associated with agricultural and urban expansion. However, the spatio-temporal analysis indicates that some areas have experienced vegetation recovery, reflecting ongoing natural regeneration. These regenerated forests are mostly secondary, with only a few remnants of primary forest. Within them, species such as Pandanus, Arrayanes, Laureles, and Guanderas promote dense, tangled regeneration that supports a high abundance of diverse taxa. However, this dynamic may further threaten morphologically cryptic lineages, such as those within the *Pristimantis myersi* group, whose diversity remains poorly understood and largely excluded from current conservation frameworks. We therefore recommend implementing restoration strategies in areas of high biological diversity to ensure the long-term survival of the species inhabiting this province one of the richest in amphibian diversity in Ecuador (Díaz-García et al., 2020).

Finally, it is important to note that the diversity documented within the *Pristimantis myersi* group in Carchi is characterized by morphologically cryptic, sympatric, and syntopic species, exhibiting pronounced intraspecific variability and polymorphism (Batallas, Márquez & Guayasamin, 2025; Batallas, Vega-Yánez & Guayasamin, Unpublished data).

This evolutionary complexity challenges traditional taxonomic frameworks. In this context, while morphological and molecular tools have been essential, they are not sufficient on their own to delimit species or to understand their population dynamics (Padial et al., 2010; Jörger & Schrödl, 2013; Zaman, Ayaz & Park, 2025) also few studies demonstrate that phenotypic evidence is sufficient to diagnose families, genera, and species group levels within clade (Ospina-Sarria & Grant, 2022), but we considered that in cryptic species groups like *Pristimantis myersi* we need an integrative approach. This study showed that morphological characteristics alone do not adequately explain the differentiation between species. In the DAPC analysis, morphological traits show a high degree of overlap, with no clear separation between groups. However, when acoustic data are included, the differentiation between species becomes more evident, highlighting the importance of using multiple lines of evidence to describe cryptic species. It is imperative to integrate genetic, bioacoustics, ecological, and biogeographic knowledge to gain deeper insight into these lineages (Wijayathilaka et al., 2018; Franco-Mena et al., 2024; Abbas et al., 2025; Kassem, 2025) and to support clear conservation and management strategies, tailored to the ecological vulnerability of the ecosystems and microhabitats they occupy.

## Conclusions

We present four new species from the mountains of the high Andean (2,784 to 3,612 m) ecosystems in Carchi province, Ecuador. These ecosystems identify an extreme and cryptic diversity of species of the *Pristimantis myersi* group with a significant number of new candidate species. Herein we describe four new species of terrestrial frogs. We highlight the importance of updating the knowledge of amphibians that are restricted to this important conservation region and comment about the threats of Carchi province and its ecosystems.

## Supplemental Information

10.7717/peerj.21618/supp-1 Supplemental Information 1 Vouchers, species, locality, and measuremnts of *Pristimantis cantrix* sp. nov., *Pristimantis carchi* sp. nov., *Pristimantis levi* sp. nov., and *Pristimantis marquezi* sp. nov. Acronym ZSFQ = Museo de Zoología Universidad San Francisco de Quito.

10.7717/peerj.21618/supp-2 Supplemental Information 2 Differences between the new species and morphologically similar taxa of the *Pristimantis myersi* group.

10.7717/peerj.21618/supp-3 Supplemental Information 3 Color variation in life of *Pristimantis cantrix* sp. nov. Photographs in lateral, dorsal, and ventral view. Photographs by Diego Batallas.

10.7717/peerj.21618/supp-4 Supplemental Information 4 Color variation in life of *Pristimantis carchi* sp. nov. Photographs in lateral, dorsal, and ventral view. Photographs by Diego Batallas.

10.7717/peerj.21618/supp-5 Supplemental Information 5 Color variation in life of (A) *Pristimantis levi* sp. nov and (B) *Pristimantis marquezi* sp. nov. Photographs in lateral, dorsal, and ventral view. Photographs by Diego Batallas.

10.7717/peerj.21618/supp-6 Supplemental Information 6 Variation in ethanol of *Pristimantis cantrix* sp. nov. Photographs in lateral, dorsal, and ventral view. Photographs by Diego Batallas.

10.7717/peerj.21618/supp-7 Supplemental Information 7 Variation in ethanol of *Pristimantis carchi* sp. nov. Photographs in lateral, dorsal, and ventral view. Photographs by Diego Batallas.

10.7717/peerj.21618/supp-8 Supplemental Information 8 Variation in ethanol of *Pristimantis levi* sp. nov. Photographs in lateral, dorsal, and ventral view. Photographs by Diego Batallas.

10.7717/peerj.21618/supp-9 Supplemental Information 9 Variation in ethanol of *Pristimantis marquezi* sp. nov. Photographs in lateral, dorsal, and ventral view. Photographs by Diego Batallas.

## Additional Information and Declarations

### Field Study Permissions

The following information was supplied relating to field study approvals (*i.e*., approving body and any reference numbers):

All specimen handling and collection were conducted under permits issued by the Ministerio del Ambiente y Energía.

### Data Availability

The following information was supplied regarding data availability:

The data is available in the Supplemental Files.

### New Species Registration

The following information was supplied regarding the registration of a newly described species:

- Publication LSID: urn:lsid:zoobank.org:pub:237DE9DD-C268-4400-9F77-CB3372ED9548

- *Pristimantis cantrix* sp. nov LSID. urn:lsid:zoobank.org:act:20F3BD35-F442-459A-BBAE-D8C4352B6EE9

- *Pristimantis carchi* sp. nov LSID. urn:lsid:zoobank.org:act:3148008B-EFE2-4146-B5A6-F1702444135C

- *Pristimantis levi* sp. nov LSID. urn:lsid:zoobank.org:act:19332353-1131-421C-86B8-AD0E79E9986A

- *Pristimantis marquezi* sp. nov LSID. urn:lsid:zoobank.org:act:4472300F-D4EA-4A64-8571-249E3C3C1077.
