# Supplementary material for: Four new species of Pristimantis myersi group (Anura: Craugastoridae) from Carchi, Ecuador: cryptic diversity in unique and threatened high-Andean ecosystems
Source: PeerJ. 2026 Aug 26;14:e21618. doi: 10.7717/peerj.21618 (PMC13526328; doi:10.7717/peerj.21618)
Supplement: Supplementary file 1 [file peerj-14-21618-s001.docx]

**Appendix S1.** Vouchers, species, locality, and measuremnts of *Pristimantis cantrix* sp. nov., *Pristimantis carchi* sp. nov., *Pristimantis levi* sp. nov., and *Pristimantis marquezi* sp. nov. Acronym ZSFQ = Museo de Zoología Universidad San Francisco de Quito.

| **Museum Number** | **Specie** | **Locality** | **Sex** | **SVL** | **HL** | **HW** | **IOD** | **UEW** | **EL** | **END** | **TY** | **IND** | **UAL** | **FLL** | **HAL** | **Fin IL** | **Fin IIDL** | **Fin IIIDW** | **THL** | **TL** | **FL** | **Toe IVDW** |
| --- | --- | --- | --- | --- | --- | --- | --- | --- | --- | --- | --- | --- | --- | --- | --- | --- | --- | --- | --- | --- | --- | --- |
| ZSFQ 4551 | *Pristimantis* *cantrix* sp. nov. | Cerro la bretaña | H | 21,2 | 8,9 | 9,4 | 2,7 | 1,8 | 2,6 | 2,2 | 1,4 | 2,4 | 4,4 | 5,6 | 6,1 | 3,1 | 3,2 | 0,9 | 9,1 | 8,8 | 10,0 | 0,8 |
| ZSFQ 4552 | *Pristimantis* *cantrix* sp. nov. | Cerro la bretaña | M | 13,7 | 6,0 | 5,7 | 1,9 | 1,7 | 1,9 | 1,2 | 1,1 | 1,8 | 2,9 | 3,6 | 4,1 | 2,0 | 2,2 | 0,5 | 6,5 | 6,6 | 6,8 | 0,6 |
| ZSFQ 4553 | *Pristimantis* *cantrix* sp. nov. | Cerro la bretaña | M | 11,8 | 5,2 | 5,1 | 1,7 | 1,2 | 1,7 | 1,1 | 0,9 | 1,6 | 2,7 | 3,2 | 3,5 | 1,7 | 1,7 | 0,5 | 5,8 | 5,9 | 6,0 | 0,5 |
| ZSFQ 4554 | *Pristimantis* *cantrix* sp. nov. | Cerro la bretaña | M | 11,6 | 5,4 | 5,1 | 1,6 | 1,4 | 1,7 | 1,2 | 1,1 | 1,6 | 2,7 | 3,0 | 3,6 | 1,8 | 1,9 | 0,4 | 5,7 | 5,5 | 5,9 | 0,5 |
| ZSFQ 4555 | *Pristimantis* *cantrix* sp. nov. | Cerro la bretaña | H | 20,7 | 8,5 | 8,6 | 2,7 | 2,1 | 2,5 | 1,7 | 1,4 | 2,2 | 4,5 | 5,3 | 5,8 | 2,9 | 3,0 | 0,8 | 8,9 | 8,8 | 10,3 | 0,8 |
| ZSFQ 4556 | *Pristimantis* *cantrix* sp. nov. | Cerro la bretaña | M | 14,1 | 6,3 | 6,2 | 2,3 | 1,6 | 1,9 | 1,2 | 1,3 | 1,9 | 2,8 | 3,5 | 3,9 | 2,0 | 2,0 | 0,5 | 6,4 | 6,4 | 7,0 | 0,6 |
| ZSFQ 4557 | *Pristimantis* *cantrix* sp. nov. | Cerro la bretaña | M | 13,1 | 5,3 | 5,2 | 1,9 | 1,4 | 1,9 | 1,2 | 1,1 | 1,5 | 2,6 | 3,1 | 3,7 | 2,0 | 2,1 | 0,5 | 5,8 | 5,7 | 6,5 | 0,5 |
| ZSFQ 4558 | *Pristimantis* *cantrix* sp. nov. | Cerro la bretaña | M | 12,4 | 5,5 | 4,9 | 1,9 | 1,4 | 1,8 | 1,1 | 1,1 | 1,6 | 3,2 | 3,0 | 3,2 | 1,6 | 1,7 | 0,4 | 5,5 | 5,6 | 5,8 | 0,4 |
| ZSFQ 4559 | *Pristimantis* *cantrix* sp. nov. | Cerro la bretaña | M | 12,0 | 5,0 | 4,8 | 1,9 | 1,2 | 1,5 | 1,0 | 0,9 | 1,5 | 2,5 | 3,1 | 3,2 | 1,6 | 1,8 | 0,4 | 5,3 | 5,5 | 5,4 | 0,4 |
| ZSFQ 4560 | *Pristimantis* *cantrix* sp. nov. | Cerro la bretaña | H | 20,8 | 7,9 | 8,0 | 2,6 | 2,1 | 2,5 | 1,4 | 1,4 | 2,4 | 4,1 | 4,7 | 5,3 | 2,6 | 2,8 | 0,6 | 8,2 | 8,1 | 9,4 | 0,7 |
| ZSFQ 4562 | *Pristimantis* *cantrix* sp. nov. | Cerro la bretaña | M | 13,9 | 6,5 | 3,4 | 2,1 | 1,6 | 1,9 | 1,2 | 0,9 | 1,9 | 3,1 | 3,7 | 4,1 | 2,1 | 2,4 | 0,5 | 7,2 | 7,1 | 7,6 | 0,6 |
| ZSFQ 4563 | *Pristimantis* *cantrix* sp. nov. | Cerro la bretaña | M | 14,3 | 5,6 | 5,7 | 1,8 | 1,5 | 1,8 | 1,2 | 1,3 | 1,7 | 3,0 | 3,9 | 3,6 | 2,0 | 2,1 | 0,5 | 6,4 | 6,0 | 6,4 | 0,5 |
| ZSFQ 4564 | *Pristimantis* *cantrix* sp. nov. | Cerro la bretaña | M | 14,4 | 6,2 | 5,8 | 2,0 | 1,5 | 1,8 | 1,4 | 1,2 | 1,9 | 3,0 | 3,8 | 4,1 | 2,2 | 2,3 | 0,6 | 7,0 | 7,0 | 7,2 | 0,6 |
| ZSFQ 4566 | *Pristimantis* *cantrix* sp. nov. | Cerro la bretaña | M | 13,8 | 5,9 | 5,5 | 1,8 | 1,4 | 1,7 | 1,2 | 0,9 | 1,7 | 2,7 | 3,6 | 3,4 | 1,8 | 1,9 | 0,5 | 6,4 | 6,3 | 6,5 | 0,5 |
| ZSFQ 4567 | *Pristimantis* *cantrix* sp. nov. | Cerro la bretaña | M | 13,9 | 5,5 | 3,4 | 1,8 | 1,3 | 1,6 | 1,2 | 1,3 | 1,6 | 2,7 | 3,5 | 3,6 | 1,7 | 1,8 | 0,5 | 5,9 | 6,0 | 6,1 | 0,6 |
| ZSFQ 4568 | *Pristimantis* *cantrix* sp. nov. | Cerro la bretaña | M | 13,8 | 5,5 | 5,2 | 1,9 | 1,4 | 1,8 | 1,2 | 1,1 | 1,7 | 3,1 | 3,5 | 3,7 | 1,9 | 1,9 | 0,4 | 6,4 | 6,3 | 6,5 | 0,5 |
| ZSFQ 4569 | *Pristimantis* *cantrix* sp. nov. | Cerro la bretaña | M | 15,4 | 6,3 | 6,2 | 1,8 | 1,7 | 2,0 | 1,3 | 1,3 | 2,0 | 3,3 | 3,5 | 3,8 | 2,0 | 2,2 | 0,5 | 6,8 | 6,2 | 6,8 | 0,5 |
| ZSFQ 4575 | *Pristimantis* *cantrix* sp. nov. | Cerro la bretaña | M | 13,1 | 5,7 | 5,2 | 1,6 | 1,4 | 1,8 | 1,3 | 1,1 | 1,7 | 2,9 | 3,3 | 3,5 | 1,8 | 1,9 | 0,4 | 6,4 | 6,1 | 6,3 | 0,5 |
| ZSFQ 4466 | *Pristimantis* *cantrix* sp. nov. | Virgen negra | M | 14,4 | 5,9 | 5,9 | 1,7 | 1,4 | 1,9 | 1,4 | 1,1 | 1,8 | 3,1 | 3,4 | 3,6 | 1,9 | 2,1 | 0,5 | 6,1 | 6,8 | 6,2 | 0,5 |
| ZSFQ 4467 | *Pristimantis* *cantrix* sp. nov. | Virgen negra | M | 16,2 | 6,4 | 6,3 | 2,0 | 1,5 | 2,1 | 1,4 | 1,4 | 1,9 | 3,5 | 3,9 | 4,0 | 1,9 | 2,1 | 0,4 | 6,9 | 6,5 | 6,9 | 0,5 |
| ZSFQ 4469 | *Pristimantis* *cantrix* sp. nov. | Virgen negra | M | 15,9 | 6,3 | 6,2 | 2,2 | 1,5 | 2,0 | 1,3 | 1,3 | 2,0 | 3,3 | 3,6 | 4,1 | 2,0 | 2,1 | 0,5 | 6,4 | 6,6 | 6,9 | 0,6 |
| ZSFQ 4471 | *Pristimantis* *cantrix* sp. nov. | Virgen negra | M | 13,2 | 5,9 | 6,1 | 2,1 | 1,5 | 1,7 | 1,3 | 1,3 | 1,8 | 2,9 | 3,3 | 3,7 | 1,8 | 1,9 | 0,5 | 5,6 | 5,9 | 6,5 | 0,6 |
| ZSFQ 4472 | *Pristimantis* *cantrix* sp. nov. | Virgen negra | H | 19,9 | 7,8 | 7,7 | 2,7 | 1,6 | 1,9 | 1,7 | 1,3 | 2,2 | 3,8 | 4,8 | 5,3 | 2,6 | 2,8 | 0,7 | 8,0 | 8,4 | 9,3 | 0,7 |
| ZSFQ 4473 | *Pristimantis* *cantrix* sp. nov. | Virgen negra | M | 12,7 | 5,8 | 5,4 | 1,8 | 1,4 | 1,8 | 1,4 | 1,3 | 1,8 | 3,3 | 3,6 | 3,4 | 1,8 | 2,0 | 0,5 | 5,9 | 5,9 | 6,2 | 0,5 |
| ZSFQ 4475 | *Pristimantis* *cantrix* sp. nov. | Virgen negra | M | 14,5 | 6,1 | 5,8 | 2,0 | 1,6 | 1,8 | 1,4 | 1,4 | 1,8 | 3,3 | 3,6 | 4,0 | 1,9 | 2,1 | 0,5 | 6,2 | 6,3 | 6,6 | 0,5 |
| ZSFQ 4508 | *Pristimantis* *cantrix* sp. nov. | Loma la esperanza | M | 12,0 | 5,4 | 4,8 | 1,6 | 1,3 | 1,6 | 1,0 | 0,9 | 1,4 | 3,3 | 3,0 | 3,3 | 1,7 | 1,8 | 0,5 | 5,8 | 5,9 | 6,0 | 0,5 |
| ZSFQ 4509 | *Pristimantis* *cantrix* sp. nov. | Loma la esperanza | M | 12,7 | 5,5 | 5,3 | 1,8 | 1,5 | 1,7 | 1,0 | 1,1 | 1,6 | 3,1 | 3,3 | 3,6 | 1,8 | 2,1 | 0,5 | 6,0 | 6,5 | 6,6 | 0,6 |
| ZSFQ 4510 | *Pristimantis* *cantrix* sp. nov. | Loma la esperanza | M | 12,4 | 5,2 | 5,3 | 1,8 | 1,4 | 1,6 | 1,1 | 1,0 | 1,5 | 2,9 | 3,1 | 3,4 | 1,8 | 2,1 | 0,5 | 5,9 | 6,0 | 6,2 | 0,5 |
| ZSFQ 4511 | *Pristimantis* *cantrix* sp. nov. | Loma la esperanza | H | 20,5 | 8,3 | 9,0 | 2,4 | 1,8 | 2,4 | 2,0 | 1,5 | 2,3 | 4,9 | 5,5 | 5,7 | 3,4 | 3,6 | 0,8 | 8,7 | 9,2 | 9,9 | 0,9 |
| ZSFQ 4512 | *Pristimantis* *cantrix* sp. nov. | Loma la esperanza | M | 13,4 | 5,4 | 5,5 | 1,9 | 1,3 | 1,8 | 1,2 | 1,3 | 1,8 | 2,9 | 3,7 | 3,8 | 1,9 | 2,1 | 0,5 | 6,3 | 6,5 | 6,9 | 0,6 |
| ZSFQ 4513 | *Pristimantis* *cantrix* sp. nov. | Loma la esperanza | M | 12,8 | 5,4 | 5,4 | 1,8 | 1,2 | 1,7 | 1,2 | 1,1 | 1,6 | 2,5 | 3,2 | 3,7 | 1,8 | 2,0 | 0,6 | 5,9 | 6,3 | 6,5 | 0,6 |
| ZSFQ 4514 | *Pristimantis* *cantrix* sp. nov. | Loma la esperanza | M | 14,7 | 6,2 | 6,0 | 1,9 | 1,7 | 1,9 | 1,4 | 1,1 | 1,8 | 3,0 | 3,6 | 3,6 | 2,0 | 2,1 | 0,5 | 6,6 | 6,4 | 6,5 | 0,6 |
| ZSFQ 4517 | *Pristimantis* *cantrix* sp. nov. | Loma la esperanza | M | 13,0 | 5,3 | 4,8 | 1,8 | 1,3 | 1,8 | 1,1 | 0,9 | 1,6 | 2,8 | 3,1 | 2,9 | 1,5 | 1,7 | 0,5 | 5,9 | 6,3 | 5,9 | 0,6 |
| ZSFQ 4518 | *Pristimantis* *cantrix* sp. nov. | Loma la esperanza | M | 12,2 | 5,1 | 4,9 | 1,8 | 1,4 | 1,7 | 1,1 | 0,9 | 1,5 | 2,8 | 3,2 | 3,4 | 1,7 | 1,9 | 0,5 | 5,7 | 6,4 | 6,5 | 0,5 |
| ZSFQ 4520 | *Pristimantis* *cantrix* sp. nov. | Loma la esperanza | M | 13,1 | 5,4 | 5,0 | 1,7 | 1,3 | 1,7 | 1,2 | 0,9 | 1,6 | 2,7 | 3,5 | 3,6 | 1,8 | 2,0 | 0,5 | 6,1 | 6,4 | 6,5 | 0,5 |
| ZSFQ 4521 | *Pristimantis* *cantrix* sp. nov. | Loma la esperanza | M | 12,6 | 5,4 | 5,3 | 1,7 | 1,3 | 1,7 | 1,1 | 1,1 | 1,6 | 3,0 | 3,6 | 3,8 | 1,9 | 2,0 | 0,5 | 6,1 | 6,6 | 6,7 | 0,6 |
| ZSFQ 4522 | *Pristimantis* *cantrix* sp. nov. | Loma la esperanza | M | 13,7 | 6,4 | 6,1 | 1,8 | 1,6 | 2,0 | 1,3 | 1,3 | 1,8 | 3,0 | 3,6 | 3,8 | 1,9 | 2,2 | 0,5 | 6,8 | 6,8 | 7,0 | 0,6 |
| ZSFQ 4523 | *Pristimantis* *cantrix* sp. nov. | Loma la esperanza | M | 12,7 | 5,3 | 2,3 | 1,7 | 1,5 | 1,7 | 1,1 | 1,1 | 1,7 | 2,6 | 3,5 | 3,8 | 1,9 | 2,1 | 0,5 | 5,9 | 6,2 | 6,9 | 0,6 |
| ZSFQ 4446 | *Pristimantis carchi* sp. nov. | Camino tufiño maldonado | M | 15,0 | 6,3 | 6,0 | 2,0 | 1,8 | 2,0 | 1,3 | 1,2 | 2,1 | 3,4 | 3,9 | 4,4 | 2,1 | 2,6 | 0,7 | 7,2 | 7,2 | 7,7 | 0,8 |
| ZSFQ 4447 | *Pristimantis carchi* sp. nov. | Camino tufiño maldonado | M | 16,2 | 5,9 | 6,2 | 2,0 | 1,7 | 2,0 | 1,3 | 1,4 | 1,8 | 3,3 | 3,9 | 4,0 | 1,9 | 2,3 | 0,6 | 7,5 | 7,6 | 7,3 | 0,7 |
| ZSFQ 4448 | *Pristimantis carchi* sp. nov. | Camino tufiño maldonado | M | 13,9 | 5,7 | 5,8 | 1,8 | 1,5 | 1,9 | 1,2 | 0,9 | 1,8 | 3,1 | 3,8 | 4,0 | 1,8 | 2,2 | 0,6 | 7,3 | 7,4 | 7,4 | 0,6 |
| ZSFQ 4449 | *Pristimantis carchi* sp. nov. | Camino tufiño maldonado | M | 15,7 | 5,9 | 5,7 | 2,1 | 1,4 | 2,0 | 1,3 | 1,3 | 1,9 | 3,1 | 4,1 | 4,3 | 2,1 | 2,4 | 0,7 | 7,3 | 7,3 | 7,5 | 0,7 |
| ZSFQ 4450 | *Pristimantis carchi* sp. nov. | Camino tufiño maldonado | M | 14,4 | 5,8 | 6,4 | 2,2 | 1,6 | 2,2 | 1,4 | 1,2 | 2,0 | 3,3 | 3,9 | 3,9 | 2,0 | 2,1 | 0,6 | 7,3 | 7,4 | 7,2 | 0,7 |
| ZSFQ 4451 | *Pristimantis carchi* sp. nov. | Camino tufiño maldonado | M | 14,3 | 6,1 | 5,9 | 1,8 | 1,6 | 2,0 | 1,3 | 1,1 | 1,8 | 3,7 | 3,9 | 3,9 | 2,0 | 2,3 | 0,6 | 7,0 | 7,0 | 7,3 | 0,7 |
| ZSFQ 4452 | *Pristimantis carchi* sp. nov. | Camino tufiño maldonado | M | 14,4 | 5,9 | 6,2 | 2,1 | 1,5 | 1,9 | 1,4 | 1,2 | 1,9 | 3,0 | 4,2 | 4,2 | 2,1 | 2,2 | 0,6 | 7,4 | 7,8 | 7,6 | 0,6 |
| ZSFQ 4453 | *Pristimantis carchi* sp. nov. | Camino tufiño maldonado | M | 15,2 | 5,9 | 6,3 | 1,7 | 1,7 | 2,0 | 1,4 | 1,2 | 2,0 | 3,1 | 3,7 | 4,4 | 2,2 | 2,4 | 0,8 | 7,1 | 7,5 | 7,7 | 0,8 |
| ZSFQ 4565 | *Pristimantis carchi* sp. nov. | Cerro la bretaña | M | 18,9 | 6,5 | 6,9 | 2,2 | 1,6 | 2,3 | 1,7 | 1,5 | 2,0 | 3,4 | 4,6 | 4,8 | 2,4 | 2,7 | 0,9 | 8,8 | 9,2 | 9,1 | 0,8 |
| ZSFQ 4570 | *Pristimantis carchi* sp. nov. | Cerro la bretaña | M | 13,5 | 5,2 | 5,6 | 1,9 | 1,6 | 1,9 | 1,2 | 1,1 | 1,8 | 3,1 | 3,9 | 4,3 | 2,1 | 2,2 | 0,7 | 7,7 | 8,2 | 7,9 | 0,7 |
| ZSFQ 4572 | *Pristimantis carchi* sp. nov. | Cerro la bretaña | M | 15,6 | 6,1 | 6,2 | 2,3 | 1,6 | 2,1 | 1,6 | 1,2 | 2,0 | 3,3 | 4,5 | 4,6 | 2,2 | 2,5 | 0,8 | 8,3 | 8,4 | 8,4 | 0,7 |
| ZSFQ 4573 | *Pristimantis carchi* sp. nov. | Cerro la bretaña | H | 21,5 | 8,1 | 8,1 | 2,7 | 1,6 | 2,6 | 2,1 | 1,1 | 2,4 | 4,0 | 5,3 | 6,2 | 2,8 | 3,4 | 1,0 | 10,6 | 11,1 | 11,0 | 1,0 |
| ZSFQ 4576 | *Pristimantis carchi* sp. nov. | Cerro la bretaña | M | 14,7 | 5,9 | 5,7 | 2,1 | 1,6 | 1,8 | 1,2 | 1,0 | 1,8 | 3,1 | 4,1 | 4,4 | 2,2 | 2,3 | 0,7 | 7,9 | 8,5 | 8,3 | 0,7 |
| ZSFQ 4577 | *Pristimantis carchi* sp. nov. | Cerro la bretaña | M | 13,8 | 5,4 | 5,5 | 1,9 | 1,5 | 1,7 | 1,3 | 1,2 | 1,8 | 3,1 | 4,0 | 4,4 | 1,9 | 2,1 | 0,6 | 7,8 | 8,7 | 8,1 | 0,7 |
| ZSFQ 4578 | *Pristimantis carchi* sp. nov. | Cerro la bretaña | M | 12,4 | 5,9 | 5,7 | 2,1 | 1,4 | 1,8 | 1,4 | 1,1 | 1,8 | 3,2 | 4,2 | 4,2 | 2,0 | 2,3 | 0,7 | 7,9 | 8,8 | 8,3 | 0,7 |
| ZSFQ 4579 | *Pristimantis carchi* sp. nov. | Cerro la bretaña | H | 17,3 | 6,9 | 6,7 | 2,3 | 1,9 | 1,2 | 1,6 | 1,1 | 2,1 | 4,0 | 4,6 | 5,2 | 2,4 | 2,9 | 0,8 | 9,3 | 10,3 | 9,3 | 0,8 |
| ZSFQ 4476 | *Pristimantis carchi* sp. nov. | Cascadas de cartagena | H | 20,6 | 8,1 | 7,8 | 2,4 | 1,4 | 2,3 | 1,7 | 1,1 | 2,4 | 4,0 | 5,3 | 5,1 | 2,3 | 2,9 | 0,7 | 9,6 | 10,4 | 9,9 | 0,7 |
| ZSFQ 4477 | *Pristimantis carchi* sp. nov. | Cascadas de cartagena | M | 16,6 | 5,8 | 5,9 | 1,9 | 1,5 | 1,9 | 1,3 | 1,2 | 1,9 | 3,3 | 4,2 | 4,3 | 2,1 | 2,4 | 0,8 | 7,6 | 8,0 | 8,2 | 0,7 |
| ZSFQ 4478 | *Pristimantis carchi* sp. nov. | Cascadas de cartagena | M | 16,1 | 6,5 | 6,6 | 2,1 | 1,6 | 2,1 | 1,4 | 1,2 | 1,9 | 3,4 | 4,5 | 4,8 | 2,1 | 2,5 | 0,8 | 7,9 | 8,4 | 8,7 | 0,8 |
| ZSFQ 4479 | *Pristimantis carchi* sp. nov. | Cascadas de cartagena | M | 14,5 | 6,2 | 5,8 | 2,1 | 1,4 | 2,0 | 1,4 | 1,1 | 1,9 | 3,2 | 4,3 | 4,5 | 2,3 | 2,6 | 0,7 | 7,5 | 8,3 | 8,4 | 0,9 |
| ZSFQ 4480 | *Pristimantis carchi* sp. nov. | Cascadas de cartagena | M | 14,1 | 6,1 | 5,5 | 2,0 | 1,5 | 1,8 | 1,3 | 1,0 | 1,9 | 3,4 | 3,9 | 4,4 | 2,2 | 2,4 | 0,7 | 7,8 | 8,3 | 7,9 | 0,7 |
| ZSFQ 4481 | *Pristimantis carchi* sp. nov. | Cascadas de cartagena | M | 17,5 | 6,7 | 6,3 | 2,4 | 1,6 | 2,2 | 1,5 | 1,3 | 2,1 | 3,4 | 4,4 | 4,9 | 2,4 | 2,5 | 0,8 | 8,6 | 9,1 | 8,7 | 0,7 |
| ZSFQ 4482 | *Pristimantis carchi* sp. nov. | Cascadas de cartagena | M | 16,9 | 6,5 | 6,3 | 2,2 | 1,7 | 2,0 | 1,4 | 1,2 | 2,1 | 3,3 | 4,4 | 4,5 | 2,4 | 2,6 | 0,9 | 8,0 | 8,8 | 9,0 | 0,8 |
| ZSFQ 4483 | *Pristimantis carchi* sp. nov. | Cascadas de cartagena | M | 16,1 | 6,0 | 6,0 | 2,1 | 1,5 | 1,9 | 1,4 | 1,2 | 1,8 | 3,7 | 4,0 | 4,4 | 2,0 | 2,2 | 0,8 | 8,0 | 8,2 | 8,3 | 0,8 |
| ZSFQ 4484 | *Pristimantis carchi* sp. nov. | Morán | M | 13,7 | 5,8 | 5,9 | 1,9 | 1,7 | 1,9 | 1,3 | 1,1 | 1,9 | 3,1 | 3,8 | 4,5 | 1,8 | 2,2 | 0,7 | 7,8 | 8,4 | 7,7 | 0,7 |
| ZSFQ 4502 | *Pristimantis carchi* sp. nov. | Morán | M | 17,1 | 6,6 | 6,8 | 1,9 | 1,7 | 2,3 | 1,4 | 1,3 | 2,0 | 3,6 | 4,8 | 4,8 | 2,3 | 2,4 | 0,9 | 8,5 | 8,3 | 8,4 | 0,8 |
| ZSFQ 4505 | *Pristimantis carchi* sp. nov. | Morán | M | 15,9 | 6,5 | 6,7 | 2,0 | 1,6 | 1,9 | 1,4 | 1,3 | 1,9 | 3,5 | 4,6 | 4,5 | 2,0 | 2,2 | 0,8 | 7,8 | 8,0 | 8,0 | 0,9 |
| ZSFQ 4533 | *Pristimantis carchi* sp. nov. | Morán | M | 15,4 | 6,1 | 6,1 | 1,9 | 1,5 | 2,0 | 1,2 | 1,1 | 1,9 | 3,8 | 4,3 | 4,1 | 2,0 | 2,3 | 0,7 | 7,3 | 7,6 | 7,1 | 0,7 |
| ZSFQ 4534 | *Pristimantis carchi* sp. nov. | Morán | M | 15,9 | 6,4 | 5,9 | 2,3 | 1,5 | 2,3 | 1,3 | 1,2 | 2,1 | 3,4 | 4,2 | 4,4 | 2,2 | 2,3 | 0,8 | 7,5 | 7,9 | 7,8 | 0,8 |
| ZSFQ 4535 | *Pristimantis carchi* sp. nov. | Morán | M | 13,7 | 5,1 | 5,3 | 1,8 | 1,4 | 1,9 | 1,2 | 1,0 | 1,6 | 3,3 | 3,7 | 4,0 | 1,8 | 2,2 | 0,6 | 6,9 | 7,1 | 7,3 | 0,6 |
| ZSFQ 4536 | *Pristimantis carchi* sp. nov. | Morán | M | 14,8 | 6,1 | 5,6 | 1,9 | 1,5 | 2,0 | 1,3 | 1,1 | 1,8 | 3,3 | 3,9 | 4,3 | 2,0 | 2,6 | 0,7 | 7,3 | 7,8 | 7,7 | 0,8 |
| ZSFQ 4537 | *Pristimantis carchi* sp. nov. | Morán | M | 14,7 | 5,7 | 5,8 | 1,9 | 1,5 | 1,9 | 1,2 | 1,3 | 1,8 | 3,7 | 4,0 | 4,2 | 2,0 | 2,1 | 0,7 | 7,2 | 7,5 | 7,5 | 0,7 |
| ZSFQ 4538 | *Pristimantis carchi* sp. nov. | Morán | M | 14,8 | 5,7 | 5,2 | 1,8 | 1,5 | 1,8 | 1,1 | 1,0 | 1,7 | 3,0 | 3,9 | 4,0 | 1,8 | 2,2 | 0,7 | 7,4 | 7,4 | 7,5 | 0,8 |
| ZSFQ 4539 | *Pristimantis carchi* sp. nov. | Morán | M | 14,6 | 6,1 | 6,2 | 1,9 | 1,6 | 2,1 | 1,2 | 1,1 | 1,8 | 3,2 | 3,7 | 4,1 | 2,0 | 2,3 | 0,8 | 7,1 | 7,3 | 7,3 | 0,8 |
| ZSFQ 4540 | *Pristimantis carchi* sp. nov. | Morán | M | 14,7 | 5,7 | 5,2 | 2,0 | 1,5 | 2,0 | 1,3 | 1,1 | 1,6 | 2,9 | 4,2 | 4,4 | 2,0 | 2,3 | 0,7 | 7,6 | 7,8 | 7,8 | 0,7 |
| ZSFQ 4541 | *Pristimantis carchi* sp. nov. | Morán | H | 19,9 | 7,7 | 7,8 | 2,8 | 1,6 | 2,6 | 1,9 | 1,6 | 2,3 | 3,8 | 4,9 | 5,7 | 2,9 | 3,4 | 1,0 | 9,1 | 9,8 | 10,2 | 1,0 |
| ZSFQ 4485 | *Pristimantis levi* sp. nov. | Morán | M | 17,9 | 6,7 | 6,7 | 2,1 | 1,6 | 2,2 | 1,7 | 1,2 | 2,0 | 3,4 | 4,3 | 5,1 | 2,4 | 2,9 | 0,9 | 9,0 | 9,6 | 9,1 | 0,9 |
| ZSFQ 4486 | *Pristimantis levi* sp. nov. | Morán | M | 15,3 | 6,3 | 5,9 | 1,7 | 1,6 | 1,9 | 1,7 | 1,2 | 1,9 | 3,4 | 3,8 | 4,7 | 2,1 | 2,2 | 0,8 | 7,9 | 8,3 | 7,8 | 0,8 |
| ZSFQ 4488 | *Pristimantis levi* sp. nov. | Morán | M | 14,5 | 5,8 | 5,5 | 1,7 | 1,4 | 2,1 | 1,6 | 1,1 | 1,7 | 3,3 | 3,6 | 4,1 | 2,0 | 2,1 | 0,6 | 7,9 | 8,1 | 7,1 | 0,7 |
| ZSFQ 4489 | *Pristimantis levi* sp. nov. | Morán | H | 19,9 | 8,1 | 8,2 | 2,2 | 1,8 | 2,7 | 1,9 | 1,2 | 2,4 | 4,0 | 5,2 | 5,7 | 2,8 | 3,1 | 0,9 | 11,2 | 11,4 | 10,2 | 1,0 |
| ZSFQ 4497 | *Pristimantis levi* sp. nov. | Morán | M | 19,1 | 6,9 | 6,8 | 1,9 | 1,8 | 2,4 | 1,7 | 1,5 | 2,1 | 3,5 | 4,2 | 4,9 | 2,3 | 2,5 | 0,8 | 8,9 | 9,0 | 9,2 | 0,9 |
| ZSFQ 4498 | *Pristimantis levi* sp. nov. | Morán | M | 18,7 | 7,1 | 7,0 | 1,9 | 2,0 | 2,7 | 1,9 | 1,4 | 2,1 | 4,0 | 4,1 | 5,3 | 2,4 | 3,0 | 0,9 | 9,1 | 9,4 | 9,2 | 1,0 |
| ZSFQ 4500 | *Pristimantis levi* sp. nov. | Morán | M | 18,3 | 7,2 | 7,3 | 2,0 | 1,9 | 2,4 | 1,7 | 1,4 | 2,1 | 3,7 | 4,3 | 4,9 | 2,3 | 2,6 | 0,8 | 9,0 | 8,9 | 8,7 | 0,8 |
| ZSFQ 4501 | *Pristimantis levi* sp. nov. | Morán | M | 15,5 | 6,3 | 6,0 | 1,7 | 1,6 | 2,2 | 1,5 | 1,2 | 1,8 | 3,2 | 3,9 | 4,5 | 1,8 | 2,5 | 0,6 | 8,4 | 8,5 | 7,8 | 0,7 |
| ZSFQ 4426 | *Pristimantis marquezi* sp. nov. | San francisco de pioter | M | 13,7 | 5,8 | 5,6 | 1,8 | 1,5 | 1,8 | 1,4 | 1,1 | 1,8 | 3,0 | 3,9 | 3,6 | 1,9 | 2,3 | 0,6 | 6,5 | 6,8 | 7,1 | 0,7 |
| ZSFQ 4427 | *Pristimantis marquezi* sp. nov. | San francisco de pioter | M | 15,3 | 6,2 | 5,8 | 1,9 | 1,5 | 1,8 | 1,6 | 1,3 | 1,8 | 3,6 | 4,1 | 3,9 | 2,0 | 2,3 | 0,7 | 7,0 | 7,1 | 7,2 | 0,7 |
| ZSFQ 4428 | *Pristimantis marquezi* sp. nov. | San francisco de pioter | M | 15,1 | 6,0 | 5,7 | 2,1 | 1,5 | 1,9 | 1,6 | 1,2 | 1,9 | 3,6 | 4,2 | 4,1 | 2,0 | 2,3 | 0,8 | 6,9 | 7,0 | 7,5 | 0,8 |
| ZSFQ 4429 | *Pristimantis marquezi* sp. nov. | San francisco de pioter | M | 16,1 | 6,0 | 6,1 | 2,0 | 1,6 | 1,9 | 1,6 | 1,2 | 1,9 | 3,2 | 4,0 | 4,2 | 2,1 | 2,1 | 0,7 | 6,8 | 6,8 | 7,3 | 0,8 |
| ZSFQ 4430 | *Pristimantis marquezi* sp. nov. | San francisco de pioter | M | 16,6 | 6,1 | 5,7 | 1,9 | 1,4 | 1,9 | 1,7 | 1,1 | 1,9 | 3,1 | 3,8 | 4,0 | 2,0 | 2,3 | 0,8 | 6,5 | 6,8 | 7,0 | 0,8 |
| ZSFQ 4431 | *Pristimantis marquezi* sp. nov. | San francisco de pioter | M | 13,8 | 5,5 | 5,1 | 1,7 | 1,3 | 1,8 | 1,2 | 1,1 | 1,7 | 3,0 | 3,6 | 3,9 | 1,9 | 2,3 | 0,6 | 6,5 | 6,9 | 6,8 | 0,6 |
| ZSFQ 4432 | *Pristimantis marquezi* sp. nov. | San francisco de pioter | M | 15,7 | 6,0 | 5,5 | 2,0 | 1,4 | 1,8 | 1,4 | 1,3 | 1,9 | 3,4 | 3,7 | 3,7 | 1,9 | 2,3 | 0,7 | 7,2 | 7,1 | 7,0 | 0,7 |
| ZSFQ 4433 | *Pristimantis marquezi* sp. nov. | San francisco de pioter | M | 12,9 | 5,1 | 4,7 | 1,8 | 1,4 | 1,6 | 1,3 | 1,0 | 1,7 | 2,7 | 2,9 | 3,3 | 1,6 | 2,0 | 0,5 | 5,8 | 5,9 | 5,9 | 0,6 |
| ZSFQ 4434 | *Pristimantis marquezi* sp. nov. | San francisco de pioter | M | 11,3 | 4,8 | 4,4 | 1,5 | 1,1 | 1,5 | 1,0 | 0,9 | 1,5 | 2,7 | 2,9 | 2,9 | 1,4 | 1,6 | 0,5 | 5,6 | 5,7 | 5,2 | 0,5 |
| ZSFQ 4435 | *Pristimantis marquezi* sp. nov. | San francisco de pioter | M | 14,6 | 6,0 | 5,3 | 2,0 | 1,3 | 1,9 | 1,4 | 1,0 | 1,8 | 3,2 | 3,6 | 3,8 | 1,9 | 2,1 | 0,6 | 7,3 | 7,3 | 6,8 | 0,6 |
| ZSFQ 4436 | *Pristimantis marquezi* sp. nov. | San francisco de pioter | H | 21,5 | 8,2 | 8,7 | 2,8 | 1,8 | 2,4 | 2,4 | 1,2 | 2,6 | 4,6 | 5,5 | 5,9 | 2,9 | 3,3 | 0,9 | 9,5 | 10,2 | 10,9 | 1,0 |
| ZSFQ 4463 | *Pristimantis marquezi* sp. nov. | San francisco de pioter | H | 21,0 | 7,4 | 7,3 | 2,4 | 1,6 | 2,1 | 2,1 | 1,3 | 2,2 | 4,0 | 4,9 | 5,0 | 2,9 | 3,1 | 0,7 | 8,7 | 8,6 | 8,8 | 0,9 |
