# Supplementary material for: Four new species of Pristimantis myersi group (Anura: Craugastoridae) from Carchi, Ecuador: cryptic diversity in unique and threatened high-Andean ecosystems
Source: PeerJ. 2026 Aug 26;14:e21618. doi: 10.7717/peerj.21618 (PMC13526328; doi:10.7717/peerj.21618)
Supplement: Supplementary file 2 [file peerj-14-21618-s002.docx]

| Characters | *P. cantrix* sp. nov. | *P. carchi* sp. nov. | *P. gralarias* | *P. hectus* | *P. leoni* | *P. levi* sp. nov. | *P. munozi* | *P. ocreatus* | *P.marquezi* sp. nov. |
| --- | --- | --- | --- | --- | --- | --- | --- | --- | --- |
| Source | This study | This study | Guayasamin *et al.* (2018) | Lynch & Burrowes (1990) | Lynch 1976b | This study | Rojas *et al.* (2014) | Lynch (1981) | This study |
| SVL male (mm) | 11.6-16.2 (n=33) | 12.4-18.9 (n=32) | Not reported | 13.6-16.8 (n=13) | 15,5-18,3 (n=3) | 14.5-19.1 (n=7) | 14,9-19,7 (n=8) | 12.4-17.2 | 11.3-16.6 (n=10) |
| SVL female (mm) | 19.9-21.2 (n=5) | 17.3-21.5 (n=4) | 21.0 (n=1) | 19.4-22.5 (n=20) | 19.7-25.0 (n=23) | 19.9 (n=1) | Not reported | 19.6-20.2 | 21.0-21.5 (n=2) |
| Dorsal texture of skin | Finely tuberculated, some almost smooth, with scattered larger tubercles | Finely tuberculated with scattered larger tubercles | Shagreen with numerous, scattered, low tubercles | Granular, becoming tuberculated laterally | Shagreened with numerous short ridges; some tubercles on posterior back | Finely tuberculated with scattered larger tubercles | Shagreen skin, with a barely visible middorsal raphe | Low ridges, finely areolate posteriorly and laterally, smooth anteriorly | Finely tuberculated or smooth, with scattered larger tubercles |
| Heel tubercles | Tuberculate, several low-conical tubercles with an enlarged conical tubercle | Tuberculate, several low round tubercles with an enlarged conical tubercle | Small, non-conical tubercle | Small | Not enlarged | Tuberculate, several low-conical tubercles | Calcar tubercles absent, heel with few, low tubercles | Not enlarged or distinctive | Tuberculate, several low-conical tubercles with an enlarged conical tubercle |
| Tarsal tubercles | Three small conical outer tubercles present | Three small triangle or fold-like outer tubercles present | Four small, non-conical tubercles | Row of larger tubercles (than heel tubercles) along outer edge of tarsus | Present; (outer 1-3 tubercles small, conical) | 2-3 small conical outer tubercles present | Four to five fold like tubercles aligned on the outer ventrolateral surface | Not enlarged or distinctive | Three small triangular or fold-like outer tubercles present |
| Plantar tubercles | Two metatarsal bulky tubercles, inner oval to rounded, 1.3-1.5x size of subconical or oval outer tubercle | Two metatarsal bulky tubercles, inner oval 1.4-2.5x size of subconical or round outer tubercle | Inner metatarsal tubercle conspicuous, oval, 4-5x round outer metatarsal tubercle | Two metatarsal tubercles, inner oval with distal and free, 4x size of subconical outer metatarsal tubercle | Two metatarsal tubercles, outer round, subconical, 0.5x of inner, inner metatarsal tubercle a little longer than wide, not compressed | Two metatarsal tubercles, inner oval to rounded, 1.36-2.65x size of subconical outer tubercle | Outer metatarsal tubercle large, round, swollen; inner metatarsal tubercle oval, elongate, about 2x the size or the outer | Two tubercles, inner large, not compressed, 2-3x size round, non-pungent outer | Two metatarsal bulky tubercles, inner oval, 1.5-1.8x size of rounded outer tubercle |
| Dorsolateral folds | From posterior edge of eyelid to sacrum, or from posterior edge of scapula to sacrum | Postocular folds from posterior edge of eyelid to sacrum, or from posterior edge of scapula to sacrum | Not described | Prominent. In most individuals there is a pair of dorsolateral folds with little indication of other folds | Absent | From the posterior edge of scapula to sacrum. Discontinuous and irregular | Present | Low | From the posterior edge of scapula to sacrum |
| Supratympanic fold | Low | Low | Not described | Thin | If present, obscured by tubercles | Present | Absent | Not prominent | Present |
| Occipital-scapular folds | Sinusoidal, W, or )( shaped scapular ridges. In some postocular ridges as part of dorsolateral folds | Straight, sinusoidally shaped, W, \/, or )( shaped scapular ridges. From posterior edge of eyelid to scapula, in some postocular ridges as part of dorsolateral folds | Not described | W shaped formed by tubercles and ridges in most individuals | Not described | Sinusoidal, \/, or )( shaped scapular ridges. In some additional postocular V shaped ridges follow from eyelid to scapula | Present | Not described | Sinusoidal, W, or )( shaped scapular ridges. In some postocular ridges follows from eyelid tubercles to scapula, and then to sacrum, as part of dorsolateral folds |
| Discoidal fold | Present | Present | Not described | Present | Absent | Present | Not described | Prominent | Present |
| Snout shape | Acutely rounded in dorsal view, rounded in lateral profile. With a small rostral papillae at snout tip | Acutely rounded in dorsal profile, rounded in lateral profile. In some truncate at snout tip | Short, rounded in dorsal and lateral profiles. With a small papilla at tip | Short, subacuminate in dorsal view, rounded in lateral profile. With a papilla at snout tip | Short, subacuminated in dorsal view (tip feebly pointed), semitruncate in lateral profile | Short, subacuminate in dorsal view, rounded in lateral profile. Without papilla at snout tip. | Short, acuminate in dorsal view, rounded in lateral profile | Very short, round in dorsal and lateral profiles | Subacuminate or acutely rounded in dorsal view, rounded in lateral view. With small rostral papilla at snout tip |
| Canthus rostralis | Sharp weakly concave | Straight, or sharp weakly concave | Distinct, weakly concave | Sharp, weakly concave | Moderately sharp, concave | Sharp weakly concave | Weakly concave in dorsal view, nearly angular in cross-section | Rounded to obtuse | Sharp weakly concave |
| Tympanum | Evident, round (3/5 - 9/10 of eye diameter), posterodorsal slightly concealed beneath supratympanic fold | Evident, round (3/5-4/5 of eye diameter), posterodorsal slightly concealed beneath supratympanic fold | Evident | Prominent, round in males, higher than long in females, larger and round in males | Partially concealed beneath skin, higher than long, (1/3-2/5 of eye diameter), larger in males than in females | Evident, round (1/2-4/5 of eye diameter), posterodorsal slightly concealed beneath supratympanic fold | Small, but well-defined, slightly higher than long (1/2-2/5 of eye diameter). Tympanic anulus prominent anteroventrally, posterodorsally concealed beneath skin. | Concealed ( upon drying annulus visible) | Evident, round (3/5 - 4/5 of eye diameter), posterodorsal slightly concealed beneath supratympanic fold |
| Relative lengths of toes | TV shorter than TIII, extending to the medial subarticular tubercles of TIV | TV shorter than TIII, extending between medial and distal subarticular tubercles of TIV | TV about same length as TIII | Not described | Not described | TV shorter than TIII, extending to the medial, or between medial and distal subarticular tubercles of TIV | TV slightly longer than TIII, and not extending to the proximal edge of the distal subarticular tubercle of TIV | Not described | TV shorter than TIII, extending to the medial subarticular tubercles of TIV |
| Dorsal body pattern | Grey, brown or greenish, with irregular olive or orange-brown marks, and occasionally longitudinal stripes in earthy or olive shades | Pale brown to black, with irregular chocolate mahogany, or olive-yellow markings. Light grey tones on the top of tubercles and folds | Grayish brown, with black marks, flanks pale brown with an olive blotch and black marks | Gray or brown (tan or reddish-brown) with dark brown (creamy-white or black) markings. Some have a dark brown middorsal line and some also have prominent dark brown dorsolateral lines | Brown (to reddish-brown) with darker brown (dark gray, black, tan and cream) markings | Khaki grey to greenish, with irregular dark brown or bottle green markings | Grayish brown, with small and irregular black spots scattered on the dorsal surface of head, upper eyelids, and dorsum. All tubercles and folds on dorsum of head and body with reddish brown coloration. | Brown with diffuse darker brown mottling. Cream edged with reddish-brown middorsal raphe from scapula to vent present or not. Most have pale orange spots along dorsolateral folds | Terra brown to greenish or black, with irregular traffic-black or brown-beige markings over the back |
| Groin coloration | Greenish-grey to brown or yellowish-grey, with or without white, olive-yellow, or ivory spots | Olive yellow, greenish-geige, or brownish beige. In females background beige-grey to black, with or without reddish-beige spots. | Dark red | Brown, with or without cream spots/flecks | White spots and/or colorless areas (small orangish-yellow spots) | Olive yellow, reed green, or green brown | Background translucent gray, with several small immaculate white dots | Brown to dark-brown with pale spots (white with pink cast in life) | Khaki grey to dark or chocolate brown, with pearl-beige or light-ivory spots; females with salmon-pink spots |
| Ventral patterns | Greenish-grey to brown or yellowish-grey, with or without khaki-grey, olive-yellow, or platinum-grey blotches; one specimen (ZSFQ 4521) shows a brownish-grey longitudinal and transversal stripe across the middle of the venter and chest | Olive yellow, green beige, pebble grey, or terra brown. In female, some with platinum grey spots. | Black with minute white spots | Pale brown with vague brown blotching (or yellow-tan with brown flecks) | Gray to brown with cream spots (white flecks and/or small yellow spots) | Agate grey, green beige, or pebble grey background; with Olive green, green grey, chrome green, or black olive blotching | Pale orange finely punctuated of black | Dark brown, spotted with cream or not. Individuals having dorsal raphe have cream line down midline on venter and another (perpendicular) across breast | Black brown, khaki grey, or chocolate brown background, with beige grey, grey brown or brown green blotching |
| Iris coloration | Agate grey, olive green, pale green, or olive yellow; with an olive drab, black brown, or traffic black median stripe | Pebble grey, pale green, mouse grey; with a bottle green, black brown or olive drab median stripe | Black with minute golden and silver spots, and orange circunpupillary ring | Dull brown to greenish or bluish-bronze with a red median stripe | Not described | Brown grey, stone grey, or olive green; with an olive drab, black brown or brown green median stripe | Golden with fine black venation and a broad horizontal copper band, pupil ring golden | Blue gray | Pastel green to brown grey; with an olive drab, or traffic black median stripe |
